# Ultrasonication coupled to enzymatic hydrolysis of soybean okara proteins for producing bioactive and bioavailable peptides

**DOI:** 10.1016/j.crfs.2024.100919

**Published:** 2024-11-07

**Authors:** Lorenza d’Adduzio, Melissa Fanzaga, Anna Laura Capriotti, Enrico Taglioni, Giovanna Boschin, Aldo Laganà, Lukas Rueller, Josef Robert, Antje van Gemmern, Carlotta Bollati, Carmen Lammi

**Affiliations:** a Department of Pharmaceutical Sciences, University of Milan, Via Mangiagalli 25, 20133, Milan, Italy; b Department of Chemistry, Sapienza University of Rome, Piazzale Aldo Moro 5, 00185, Rome, Italy; c Fraunhofer Institute for Environmental, Safety and Energy Technology UMSICHT, 46047, Oberhausen, Germany

**Keywords:** Angiotensin-converting enzyme (ACE), Antioxidant activity, Bioactive peptides, Dipeptidyl peptidase-IV (DPP-IV), Okara, Peptide bioavailability, Soybean okara

## Abstract

This work was aimed to explore the antioxidative properties, bioavailability and the safety of bioactive peptides obtained by the enzymatic hydrolysis of ultrasound-treated (UO) and untreated (nUO) soybean okara proteins. Particularly, the peptidomic profiles of both hydrolysates were examined using an untargeted metabolomics technique for suspect screening that was specifically designed for the profiling of short-chain peptides and relied on ultra-high-performance liquid chromatography coupled to high-resolution mass spectrometry (UHPLC-HRMS) and bioinformatics.

Next, both UO and nUO hydrolysates reduce Dipeptidyl peptidase-IV (DPP-IV) enzyme activity until 39.54 ± 0.26 % and 43.29 ± 0.36 % respectively and inhibit angiotensin converting enzyme (ACE) activities by 30.54 ± 0.42 % and 30.76 ± 0.02 %, respectively. Moreover, they demonstrate to exerted antioxidant properties. Particularly, they show a comparable *in vitro* antioxidant activity but when the oxidative stress is induced by H_2_O_2_ in Caco-2 cells, UO hydrolysate is more active in lowering the levels of reactive oxygen species (ROS) and of lipid peroxidation induced of 48% and 20% respectively.

In addition, UO- and nUO-derived peptides trans-epithelial transported by human differentiated intestinal cell monolayer, were identified. Lastly, the possible hepatotoxicity of UO and nUO hydrolysates in HepG2 cells has not been observed by measuring alanine transferase (ALT) and aspartate transferase (AST) levels and cytotoxic effects.

## Highlights

• Soybean okara proteins (UO and nUO) were hydrolyzed using papain and alcalase.
• Small peptides were enriched by 3 kDa ultrafiltration after hydrolysis.
• UO and nUO hydrolysates contain bioactive peptides with DPP-IV and ACE activity.
• These hydrolysates show antioxidant properties *in vitro* and on Caco-2 cells.
• UO and nUO di- and tri-peptides are absorbed by *in vitro* intestinal cells.

## Graphical abstract

**Figure:**
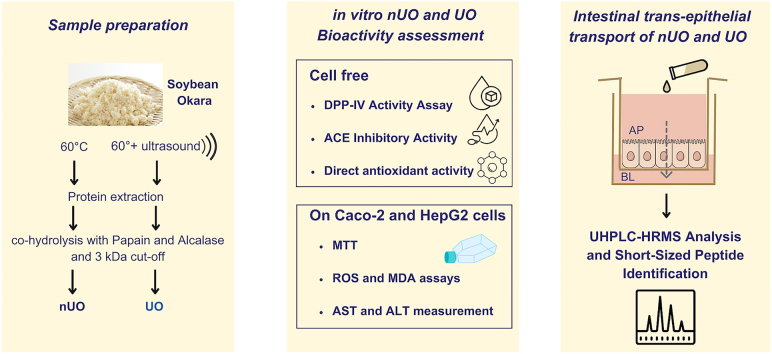

## 1 Introduction

Soybean and its by-products provide affordable protein sources that are rich in nutrients (Canaan et al., 2022). Increased soybean production worldwide has resulted in a growth in the popularity of soy-based foods (i.e., tofu, soymilk, and soy nuts) (Messina, 2016). On the other hand, significant wastes/by-products are generated during the processing of soybeans, such as soybean okara. Industries frequently underuse okara by-products, despite its high nutritional content and quantity of bioactive components, such as vitamins as Thiamine (B1), Riboflavin (B2), Niacin (B3), minerals (K, Na, Ca, Mg, Fe, Cu, Mn, and Zn) and phytochemicals (Isoflavone aglycones, Isoflavone glucosides, Malonyl glucosides, Acetyl glucosides, phytic acid and saponins) (Čech et al., 2022; Vong and Liu, 2016). In addition, it contains approximately 28%–30% protein and 8%–10% fat, mostly polyunsaturated fatty acids (Asghar et al., 2023a). Moreover, soybean okara is rich in antioxidant amino acids such as phenylalanine, tyrosine, and methionine, whose presence would justify the antioxidant activity of this food matrix (Asghar et al., 2023b; Fang et al., 2021). Because of its high protein content, it is also a great source to produce bioactive peptides, which are becoming increasingly popular for their beneficial and health-promoting qualities, even though emphasis regarding their safety issues must be placed and their safety aspects disserve deeply investigation (Quintieri et al., 2023; Wang et al., 2024). These peptides, particularly the ones derived from the enzymatic hydrolysis of plant-derived proteins, exhibit diverse physiological activities, making them promising candidates for various nutraceutical applications (Fan et al., 2022). The production of food-derived bioactive peptides can be achieved through enzymatic hydrolysis, microbial fermentation, or chemical hydrolysis. Enzymatic hydrolysis is a straightforward process that can be easily controlled and optimized to yield high-quality peptides (Suo et al., 2022). Selecting the right enzymes is essential for producing a hydrolysate rich in bioactive peptides, as smaller peptides are more likely to reach the bloodstream and target organs intact (Mora and Toldrá, 2023). Plant derived bioactive peptides have been demonstrated to exert a wide range of functional activities, including antihypertensive, antimicrobial, antidiabetic, and anti-inflammatory effects, among others, promoting human health and well-being (Görgüç et al., 2020). In several studies antioxidant peptides derived from soybean okara have been shown to possess potent scavenging abilities against reactive radicals, thereby mitigating and ameliorating oxidative damage, which is often implicated in numerous chronic diseases (Fang et al., 2021; Xie et al., 2024).

Moreover, as reported in a previous study, the application of ultrasound technology has shown prospects for improving the functional characteristics of proteins extracted from soybean okara, leading to an increase in bioactive peptides generation, possessing enhanced antioxidant activity as well (Aiello et al., 2021a). The use of innovative processing technologies, like ultrasound, results advantageous in the search for unique functional components, supported by recent studies in the field of functional compounds, that demonstrated the effectiveness of ultrasonic treatment for bettering the functioning of certain molecules or the release of bioactive peptides (Rivero-Pino et al., 2020). High-power ultrasound treatment enables the cell disintegration of raw materials and hence the effective extraction of target molecules into the surrounding liquid medium. This is caused by the process of acoustic cavitation. Here, ultrasound waves lead to pressure changes within the liquid medium leading to the formation and implosion of microbubbles. Besides mechanical shear forces, the cavitation can also cause sonochemical effects on molecules, which may lessen allergenicity and the presence of antinutritional components (Sengar et al., 2022). Therefore, in agreement with these observations, up to now, the ultrasound pre-treatment has been widely applied for improving the protein recovery yields from the food matrices and/or by-products (Chemat et al., 2011).

For the first time, this study aims to valorize and correlate the health promoting activity of soybean okara hydrolysates obtained using ultrasonication coupled to enzymatic hydrolysis. To achieve this overall objective, ultrasound-treated (UO) and untreated (nUO) soybean okara proteins were enzymatically hydrolyzed using food-grade enzymes, to obtain mixtures of enriched in bioactive peptides.

These mixtures underwent a multidisciplinary investigation aimed at comparing their composition, biological activity, bioavailability, and safety profiles between UO and nUO samples. Peptide hydrolysates (UO and nUO) were produced and applying ultrafiltration technique with a <3 KDa cut-off, fractions enriched in short-sized peptide mixtures were obtained. Indeed, their composition was investigated by a suspect screening untargeted metabolomics approach based on ultra-high-performance liquid chromatography coupled to high-resolution mass spectrometry (UHPLC-HRMS) and bioinformatics, devised explicitly for the profiling of short-chain peptides. Subsequently, the in DPP-IV and ACE inhibitory activity, as well as *in vitro* and cellular antioxidant properties of the resulting hydrolysates were assessed utilizing human intestinal Caco-2 cells. Lastly, the study investigates the effect that ultrasound pre-treatment coupled to enzymatic hydrolysis can exert on the intestinal transepithelial transport of nUO and UO hydrolysates in differentiated Caco-2 cells. In general, short-chain peptide mixture tend to be absorbed faster than medium- and/or long-chain ones by enterocytes (Aiello et al., 2019). Hence, differentiated Caco-2 cells were used as a reliable model for assessing the ability of short-chain peptide to be transported at the intestinal level, addressing delicate issues related to their stability and bioavailability.

Hence, overall, by combining high-power ultrasonication and enzymatic hydrolysis, this study provides new and innovative insight into the improved bioactivity and bioavailability of soybean okara peptides upon ultrasound pre-treatment coupled to enzymatic hydrolysis. This might have significant implications for the application of soybean okara in functional foods and nutraceuticals and may contribute to improving human health and well-being.

## 2 Materials and methods

### 2.1 Material and chemicals

All chemicals and reagents were of analytical grade and from commercial sources. Hydrochloric acid (HCl) (ACS Reagent 258148), 3-(4,5-dimethylthiazol-2-yl)-2,5- diphenyltetrazolium bromide (MTT) (Sigma-Aldrich 475989), ROS (Sigma Aldrich MAK143) and lipid peroxidantion Sigma-Aldrich (MAK568) (MDA) assay kits were from Sigma- Aldrich (St. Louis, MO, USA). Dulbecco's modified Eagle medium (DMEM) (Euroclone ECM0728L), fetal bovine serum (FBS) Euroclone ECS5000L, L-glutamine, phosphate buffered saline (PBS) (Euroclone ECB4053), penicillin/streptomycin (Euroclone ECB3001D), 24 and 96- well plates (Euroclone ET3024, ET3096) were from Euroclone (Milan, Italy). Human AST and ALT Elisa kits (Abcam ab263881, ab234578) were purchased from Abcam (Cambridge, UK). Papain and alcalase enzymes (Sigma Aldrich, Milan, Italy, P3375-25G, 3611125).

### 2.2 Okara samples preparation and hydrolysis with papain and alcalase

Experiments of ultrasound-assisted soybean okara processing were performed at Fraunhofer UMSICHT, Germany. A defined ratio of 1:2.5 of soybean okara and water (pH 6.5 ± 0.14) was heated up to the experimental temperature of 60 °C in continuous stirring. Ultrasound parameters of power input (3,5 kW), correlating oscillation amplitude (40 μm), and the treatment time of 30 s was fixed. Hence, the total energy input for all samples was 24 kJ/L. The processed samples were pressed with a screw press. The liquid phase was freeze-dried with a program for plant extract (Christ Alpha 2–4 LSC Plus (Co. Martin Christ)). These fractions of untreated soybean okara (sUO) and ultrasound-treated soybean okara (UO) were used for further analyses. Proteins from nUO and UO (liquid fraction) were extracted with a dedicated buffer (100 mM Tris−HCl/0.5 M NaCl buffer at pH 8.0) in a ratio 1:20 in continuous stirring overnight. Samples were then centrifuged (8000g; 25 min; RT) and proteins contained in the supernatant were precipitated overnight in cold acetone in a ratio of 1:4 (sample: acetone). Pelleted proteins were resuspended and Bradford experiments using a BSA standard curve were carried out to quantify the proteins present in both samples. Proteins were then hydrolyzed in optimized conditions exploiting food-grade enzymes, namely papain and alcalase. Thus, a co-hydrolysate was generated first digesting with papain (5 h; pH 7; 65 °C; 100AU/g) and then with alcalase (4 h; pH 8.5; 50 °C; 0.15 AU/g). In order to verify the efficiency of the hydrolysis, a SDS page analysis was conducted, loading the total protein extracts of both samples and the same samples at different hydrolysis time points, namely after 0, 5, and 9 h from the starting point. The degree of hydrolysis (% DH) for each hydrolysate after 0-2-5-7-9 h of hydrolysis was then identified by the o-phthaldialdehyde (OPA) method (Aiello et al., 2017). Both hydrolysates have been finally enriched in small and medium peptides trough ultrafiltration with 3 kDa centrifugal filters and samples have been then freeze-dried.

### 2.3 *In vitro* DPP-IV activity assay

The experiments were carried out in a half-volume 96-well solid plate (white) with nUO and UO hydrolysates at final concentration range of 0,5-1-2,5 mg/mL and using conditions previously optimized (Lammi et al., 2016a, Lammi et al., 2016b).

### 2.4 *In vitro* measurement of ACE inhibitory activity

To assess their ACE-inhibitory activity, the nUO and UO hydrolysates were tested as previously reported (Boschin et al., 2014a, 2014b).

### 2.5 Evaluation of the direct antioxidant activity of Okara hydrolysates

#### 2.5.1 Diphenyl-2-picrylhydrazyl radical (DPPH) assay

The DPPH assay was carried out using a conventional procedure with a slight modification (Lammi et al., 2020a, Lammi et al., 2020b). In brief, 45 μL of 0.0125 mM DPPH solution (which was dissolved in methanol) was mixed with 15 μL of nUO and UO with final concentrations of 1 and 5 mg/mL. The reaction for scavenging DPPH radicals took place in the dark at room temperature, then the absorbance was measured at 520 nm (Abramovič et al., 2018) after a period of 30 min incubation.

#### 2.5.2 2,2′-azino-bis (3-ethylbenzothiazoline-6-sulfonic acid) diammonium salt assay

The decrease of the 2,2-azino-bis-(3-ethylbenzothiazoline-6-sulfonic) acid (ABTS) radical caused by antioxidants is the basis of the Trolox equivalent antioxidant capacity (TEAC) assay (Nenadis et al., 2004). A 7 mM ABTS solution (Sigma-Aldrich, Milan, Italy) was combined with 2.45 mM potassium persulfate (1:1) to create the ABTS radical cation (ABTS+•), which was then kept for 16 h at room temperature and in the dark. The ABTS+• was diluted in 5 mM phosphate buffer (pH 7.4) to obtain a stable absorbance of 0.700 (±0.02) at 730 nm, which was employed to prepare the ABTS reagent (Tai et al., 2016). For the experiment, 70 μL of diluted ABTS+• was mixed with 5 μL of nUO and UO at final concentrations of 0.05, 0.1, 0.5, 1 and 5 mg/mL. After 30 min of 30 °C incubation, the absorbance at 730 nm was measured using a microplate reader Synergy H1 (Biotek).

#### 2.5.3 FRAP assay

The FRAP assay measures a sample's capacity to convert ferrous ions (Fe^2+^) from ferric ions (Fe^3+^) (Benzie and Devaki, 2017). Thus, 70 μL of FRAP reagent was combined with 5 μL of nUO and UO at the final concentrations of 0.5, 1 and 5 mg/mL. The FRAP reagent was generated by combining 1.3 mL of 0.3 M acetate buffer (pH 3.6), 1.3 mL of 20 mM FeCl_3_ × 6 H_2_O, and 1.3 mL of a 10 mM TPTZ (Sigma-Aldrich, Milan, Italy) solution in 40 mM HCl. The absorbance at 595 nm was measured after the microplate was incubated for 30 min at 37 °C. Microplate reader Synergy H1 (Biotek) was used to record absorbances.

### 2.6 Cell culture

Caco-2 cells and HepG2 cells, obtained from INSERM (Paris, France) bought from ATCC (HB- 8065, ATCC from LGC Standards, Milan, Italy) were routinely sub-cultured following a previously optimized protocol (Lammi et al., 2016a, Lammi et al., 2016b) and maintained at 37 °C in a 5% CO_2_ atmosphere in DMEM containing 25 mM of glucose, 3.7 g/L of NaHCO_3_, 4 mM of stable L-glutamine, 1% non-essential amino acids, 100 U/L of penicillin and 100 μg/L of streptomycin (complete medium), supplemented with 10% heat-inactivated FBS.

### 2.7 3-(4,5-Dimethylthiazol-2-yl)-2,5-diphenyltetrazolium bromide (MTT) assay

Experiments were conducted following the standard procedure (Ghasemi et al., 2021). Briefly, 3 × 10^4^ Caco-2 cells/well or HepG2 cells were cultured in 96-well plates and treated with 1–10 mg/mL of nUO and UO and/or vehicle (H_2_O) in complete growth medium for 48 h at 37 °C in a 5% CO_2_ atmosphere. Then, culture media were discarded, 3-(4,5-dimethylthiazol-2-yl)-2,5-diphenyltetrazolium bromide (MTT solution) (5 mg/mL in PBS) was added and the plate was incubated for 2h. After that, MTT solution was removed and 100 μL/well of lysis buffer were added, the plate was shaken for 10 min and the absorbance ridden at 575 nm using the Synergy H1 fluorescence plate reader (Biotek, Bad Friedrichshall, Germany).

### 2.8 Fluorometric intracellular ROS assay

30.000 Caco-2 cells were seeded in growth media for the whole night on a black 96-well plate. The following day, the medium was eliminated, then 50 μL of Master Reaction Mix and 50 μL of complete DMEM were added to each well, and the cells were incubated for 1 h in the dark at 37 °C and 5% CO_2_. After adding the nUO and UO to achieve the desired concentrations of 5.0 mg/mL, the mixture was incubated for a full day at 37 °C. To produce reactive oxygen species (ROS), cells were exposed to 1.0 mM H_2_O_2_ for 60 min at 37 °C in the dark. Fluorescence signals (ex./em. 490/525 nm) were then recorded using a Synergy H1 microplate reader (Biotek).

### 2.9 Lipid peroxidation (MDA) assay

After being seeded in a 24-well plate, Caco-2 cells (2.5 × 10^5^ cells/well) were treated with the nUO and UO for 24 h at 37 °C in an atmosphere of 5% CO_2_. After incubating with H_2_O_2_ at a concentration of 1 mM or with vehicle (H_2_O) for 1 h, the cells were collected and homogenized in 150 μL of ice-cold MDA lysis solution that contained 3 μL of butylated hydroxytoluene (BHT). Samples were centrifuged at 13,000 g for 10 min, then each vial containing 100 μL of samples was filled with 300 μL of the TBA solution to generate the MDA-TBA adduct. The vials were then incubated at 95 °C for 60 min, then cooled to room temperature for 10 min in ice. Each reaction mixture was pipetted into a transparent 96-well plate containing 100 μL for measurement. The absorbance was measured at 532 nm using the Synergy H1 microplate reader (Biotek).

### 2.10 Caco-2 cell culture and differentiation

Caco-2 cells were grown in accordance with an earlier technique (Lammi et al., 2020a, Lammi et al., 2020b). To facilitate the establishment of a confluent cell monolayer, cells were seeded on a Transwell at a density of 3.5 × 10^5^ cells/cm^2^ in complete medium supplemented with 10% FBS in both the AP and BL compartments for a period of 2 days. Cells were seeded, and on day three, they were moved to an FBS-free medium in the AP compartment. They were then allowed to differentiate for 18–21 days, with three weekly medium changes in between (Lammi et al., 2016a, Lammi et al., 2016b). Transepithelial electrical resistance (TEER) of differentiated Caco-2 cells was measured at 37 °C using the Millicell voltmeter device (Millipore Co., Billerica, MA, USA) both before and after the transport experiments for monitoring the integrity of the cell monolayers.

### 2.11 Trans-epithelial transport experiments

TEER measurement was used to verify the integrity and differentiation of the cell monolayer prior to transport experiments. According to previously published conditions (Lammi et al., 2021a), nUO and UO trans-epithelial transit was assessed in differentiated Caco-2 cells in transport buffer solution (137 mM NaCl, 5.36 mM KCl, 1.26 mM CaCl_2_, and 1.1 mM MgCl_2_, 5.5 mM glucose). The apical (AP) solutions were kept at pH 6.0 (buffered with 10 mM morpholinoethane sulfonic acid) and the basolateral (BL) solutions were kept at pH 7.4 (buffered with 10 mM N-2-hydroxyethylpiperazine-N-4-butanesulfonic acid) in order to replicate the pH conditions found in vivo in the small intestinal mucosa. Before the transport assay, cells were equilibrated in HBSS for 15 min at 37 °C. In the AP compartment with the AP transport solution (500 μL) and the BL compartment with the BL transport solution (700 μL), nUO and UO (5 mg/mL) were added. All BL and AP solutions were collected after 2 h of absorption experiment carried out at 37 °C, and they were kept at - 80 °C before analysis. Transport experiments were performed in duplicate.

### 2.12 UHPLC-HRMS analysis and short-sized peptide identification

Short peptides were analyzed by Vanquish binary pump H (Thermo Fisher Scientific, Str. Rivoltana—Rodano, Milan, Italy) coupled to a hybrid quadrupole—Orbitrap mass spec-trometer Q Exactive (Thermo Fisher Scientific, Str. Rivoltana—Rodano, Milan, Italy) using a heated ESI source operating in positive ion mode. The mass-spectrometric strategy was developed as previously documented (Cerrato et al., 2020).

### 2.13 Free amino acids (a.a.) quantification

The sample was diluted 1:1 with 0.2 N lithium citrate buffer pH 2.2. The solution was filtered on 0.2 μm membrane filter (Millipore, Milford MA, USA) and analyzed by ion exchange chromatography using an amino acid analyser Biochrom 30+ (Erreci, Milan, Italy) as described by Hogenboom et al. (Hogenboom et al., 2017). The content of individual amino acids was calculated using five-level calibration curves.

### 2.14 Alanine aminotransferase (ALT) and aspartate aminotransferase (AST) quantification in HepG2 cells supernatants

Levels of ALT and AST were determined in HepG2 cells supernatant using ELISAs assays according to the manufacturer's instructions. 30.000 cells were cultured for the quantification of AST levels, while 50.000 cells were seeded to quantify ALT levels (96 multiwell plates) in the presence or absence of nUO and UO hydrolysates**.** The ELISA kit for ALT, AST was obtained from Abcam (Cambridge, UK). All measurements were performed in triplicate and the experiments were repeated twice.

### 2.15 Statistical analysis

Every measurement was done in triplicate, and the results were reported as the mean ± standard deviation (s.d.), with *p*-values less than 0.05 being regarded as significant. Dunnett's and Tukey's post-test were conducted after ONE and two-way ANOVA statistical analyses (GraphPad Prism 9, GraphPad Software, La Jolla, CA, USA).

## 3 Results

### 3.1 Soybean okara hydrolysates production and peptidomic characterization

Proteins from untreated soybean okara (nUO) and ultrasound-treated okara (UO) (liquid fraction) were extracted with a previously optimized buffer (100 mM Tris−HCl/0.5 M NaCl buffer at pH 8.0) (Aiello et al., 2021a) and they were precipitated in cold acetone and resuspended to be further hydrolyzed. Bradford experiments were conducted to quantify the proteins present in each sample, defining a concentration of 7.18 ± 0.013 mg/mL in UO and 2.57 ± 0.16 mg/mL in nUO, confirming that the ultrasound pretreatment of the starting material can enhance the protein extraction yield (Aiello et al., 2021a).

**Fig. 1:**
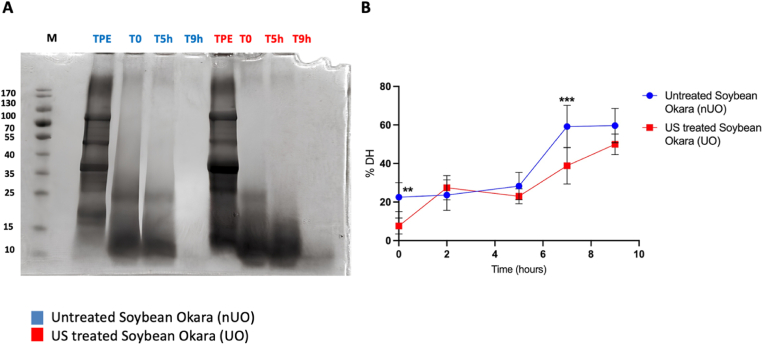
Digestion efficiency of nUO and UO total protein extract (A) and degree of hydrolysis (DH) trend (B). SDS-PAGE analysis of papain/alcalase hydrolysates samples at different hydrolysis time points; TPE: total protein extract. The data are represented as the means ± s.d. of three independent experiments. All the data sets have been analyzed by Two-way ANOVA. Not significant differences are not shown, (∗∗) *p* < 0.01, (∗∗∗) *p* < 0.001.

UO and nUO soybean okara proteins were hydrolyzed by employing two food-grade enzymes, alcalase and papain, two endopeptidases, each with distinct cleavage characteristics, for enzymatic hydrolysis. Papain is a cysteine protease preparation, produced from latex of papaya fruit, that cleaves peptide bonds within hydrophobic areas, encompassing amino acids alanine, valine, leucine, isoleucine, phenylalanine, tryptophane, and tyrosine, alcalase is a protease preparation of bacterium Bacillus licheniformis, that targets peptide bonds involving phenylalanine, tyrosine, tryptophane, and lysine carboxyl groups. UO and nUO co-hydrolysates, respectively, were generated in optimized conditions first digesting with papain and then with alcalase enzyme (Bartolomei et al., 2022). To verify the efficiency of the hydrolysis, a SDS page analysis was conducted, loading the total protein extracts (TPE) of both samples and the same samples at different hydrolysis time points, namely after 0, 5, and 9 h from the starting point (Fig. 1A). The SDS-PAGE clearly shows that, differently from TPE, in the nUO and UO digested samples the proteins fraction was completely hydrolyzed during the enzymatic process. In fact, the % DH for each hydrolysate after 0-2-5-7-9 h of hydrolysis was then identified by the o-phthaldialdehyde (OPA) method and the results show that the % DH reached the 59.7 ± 8.9% in nUO, and the 50% ± 5.3% in the UO after 9 h of hydrolysis (Fig. 1B).

Both nUO and UO containing short-chain peptide mixture were subjected to a suspect screening approach on UHPLC-HRMS instrumentation for assessing the effect of ultrasound on the peptide composition. More in details, the suspect screening approach was based on using inclusion lists in the mass spectrometric method that bypasses the limitations of data dependent acquisition mode when comprehensive lists of the analytes are available (Cerrato et al., 2022a). Moreover, the data processing workflow allowed extracting the m/z from the raw data files, aligning the features in the different samples, removing compounds present in the black sample, predicting the molecular formulas from the accurate masses and isotopic patterns, and associating the predicted formula to those of the short peptide sequences listed in the short peptide databases that were also employed for data acquisition. After careful manual validation of the putative peptides based on the peculiar short peptide fragmentation pathways, 316 and 315 short-chain peptides were tentatively isolated in nUO and UO, respectively. Table S1 reports, for short-chain peptides identified in both samples, detailed data on the tentatively identified sequences, including retention time, proposed formula, experimental m/z, MS accuracy, and primary diagnostic product ions. Since Leu and Ile cannot be distinguished by MS/MS (MS3 experiments are needed) (Cerrato et al., 2022b), the nomenclature Xle was employed throughout the manuscript and Supplementary Materials for indicating either Leu or Ile in a peptide sequence. Interestingly, no significant differences between nUO and UO peptide mixtures were observed. In nUO, of the 316 annotated short peptides, 150 (47.5%) were dipeptides and 166 (52.5%) were tripeptides with a molecular weight in the range 172.1–498.2 Da; in UO, of the 315 annotated short peptides, 147 (46.7%) were dipeptides and 168 (53.3%) were tripeptides.

**Fig. 2:**
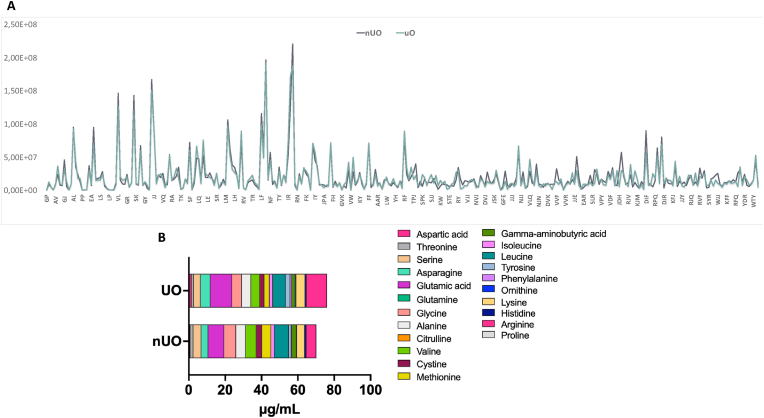
Distribution of short-sized chain peptides of nUO and UO obtained after the co-digestion using both alcalase and papain. On the x axis is shown the peptide sequences and on the y axis signal intensity (A). Free amino acids (a.a.) profile of nUO and UO (B).

Fig. 2A clearly indicated that the co-digestion using both alcalase and papain, is efficient approach for generating high amount of short-sized chain peptides, while the use of ultrasonication has no significant effect in generating new peptide species, but the use of the ultrasonication as pretreatment technique of the okara proteins led to a modification of the relative abundance of specific peptides much more than others.

The free amino acids (a.a.) quantification (Fig. 2B) suggested that ultrasound pre-treatment slightly but significantly increase the free a. a. contents in UO sample compared to nUO (*p*<∗∗∗∗). Notably, the total nUO a. a. content is 70.05 ± 0.47 μg/mL and the total UO a. a. content amount to 76.10 ± 0.26 μg/mL. Interestingly, the proline and glutamine were not identified in both samples, whereas asparagine, aspartic acid, glutamic acid, tyrosine, arginine and the essential a. a. phenylalanine are increased in UO sample vs nUO, whereas essential a. a. profile quantification suggested that leucine, methionine, threonine and valine decrease moderately in UO sample.

### 3.2 Biochemical investigation of nUO and UO hydrolysates DPP-IV and ACE inhibitory activities

#### 3.2.1 nUO and UO hydrolysates inhibit *in vitro* DPP-IV and ACE activity

**Fig. 3:**
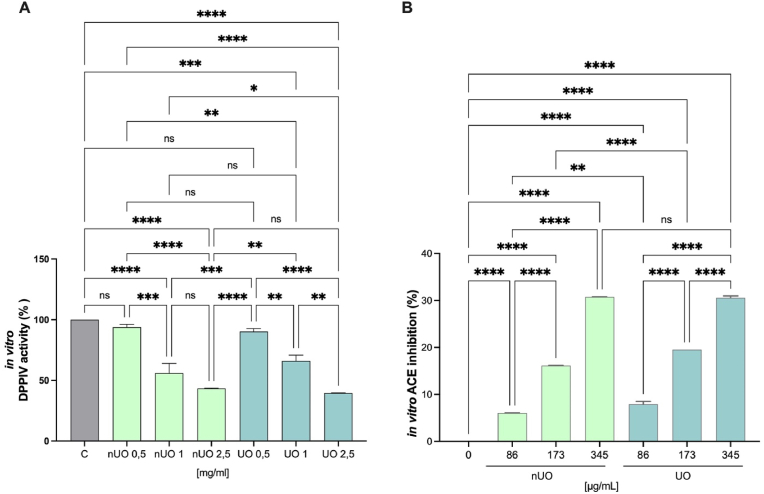
Evaluation of the *in vitro* DPP-IV inhibitory (A) and ACE-inhibitory (B) effects of nUO and UO hydrolysates. Bars represent the sd of three independent experiments in duplicate. ns. not significant, (∗) *p* < 0.05 (∗∗), *p* < 0.01, (∗∗∗) *p* < 0.001, (∗∗∗∗) *p* < 0.0001.

To assess the ability of nUO and UO hydrolysates to modulate DPP-IV activity, *in vitro* experiments were performed using the purified recombinant DPP-IV enzyme. Fig. 3A shows that both nUO and UO hydrolysates tested at 0.5, 1 and 2.5 mg/mL drop *in vitro* DPP-IV activity in a dose dependent manner. nUO by 93.87 ± 2.416%, 55.84 ± 8.10% and 43.29 ± 0.36% and UO by 90.21 ± 2.534%, 66.00 ± 4.845%, 39.54 ± 0.2611% at 0.5, 1 and 2.5 mg/mL, respectively.

In parallel, the *in vitro* ACE activity inhibition of both hydrolysates was confirmed, as shown in Fig. 3B. In particular, nUO inhibits ACE activity *in vitro* by 6.01 ± 0.07%, 16.12 ± 0.07% and 30.76 ± 0.02%, at 86, 173, 345 μg/mL, respectively, whereas UO reduced the enzymatic activity by 7.91 ± 0.58%, 19.52 ± 0.1%, 30.54 ± 0.42%, at the same concentrations.

### 3.3 Direct nUO and UO hydrolysates antioxidant activity

#### 3.3.1 Direct radical scavenging activity of nUO and UO hydrolysates by DPPH assay

**Fig. 4:**
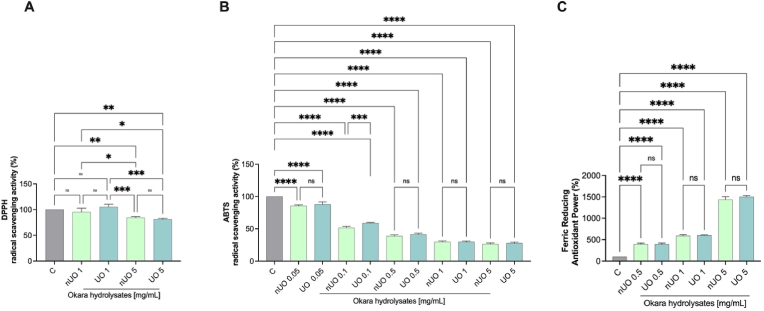
*In vitro* direct nUO and UO Hydrolysates antioxidant activity. *In vitro* radical scavenging activity of nUO and UO hydrolysates (A–B) and FRAP (C) assays, respectively. Data represent the mean ± s.d. of six determinations performed in triplicate. All the data sets have been analyzed by One-way ANOVA followed by Tukey's post-hoc test. Ns: not significative, (∗) *p* < 0.05, (∗∗) *p* < 0.01, (∗∗∗) *p* < 0.001, (∗∗∗∗) *p* < 0.0001. C: control.

The DPPH radical scavenging activity of both okara hydrolysates has been evaluated at the concentrations of 1.0 and 5.0 mg/mL. Fig. 4A shows that nUO hydrolysate and UO hydrolysate scavenged the DPPH radical by 15.96 ± 2.53% and 18.88 ± 1.83% at the concentration of 5.0 mg/mL, respectively, while when tested at lower concentration (1.0 mg/mL) both hydrolysates were not able to scavenge DDPH radical significantly versus control condition.

#### 3.3.2 Direct radical scavenging activity of nUO and UO hydrolysates by ABTS assay

To further confirm the direct antioxidant property, the ABTS assay was performed. ABTS radical scavenging activity of both the okara hydrolysates was determined at 0.05, 0.1, 0.5, 1.0 and 5.0 mg/mL, demonstrating to have a dose dependent activity in both cases. nUO scavenged the ABTS radical by 14.45 ± 1.443% at 0.05 mg/mL, 47.38 ± 1.25% at 0.1 mg/mL, 61.16 ± 1.7% at 0.5 mg/mL, 70.26 ± 1.457% at 1.0 mg/mL and 73.41 ± 1.512% at 5 mg/mL, while UO hydrolysate scavenged the ABTS radical by 12.35 ± 3.891% at 0.05 mg/mL, 41.10 ± 0.818% at 0.1 mg/mL, 58.71 ± 2.06% at 0.5 mg/mL, 70.08 ± 1.195% at 1 mg/mL, and by 72.18 ± 1.552% at 5 mg/mL. Data demonstrate that both hydrolysates are active even at low concentration with almost equal and comparable activity (Fig. 4B).

#### 3.3.3 Ferric-reducing antioxidant power (FRAP) activity

The FRAP assay was carried out, evaluating the ability of samples to reduce ferric ion (Fe3+) into ferrous ion (Fe^2+^). As shown in Fig. 4C, nUO and UO hydrolysates increased the FRAP radical by 396.6 ± 25% and 394.9 ± 27.9% at 0.5 mg/mL, respectively. When assayed at 1.0 mg/mL, nUO and UO improved the FRAP levels by 590 ± 29.3% and 603.4 ± 9.6%, respectively. Finally, nUO and UO hydrolysates, tested at 5.0 mg/mL, improved the FRAP by 1437 ± 67% and 1502 ± 31.6% respectively.

### 3.4 Ultrasound treated soybean okara hydrolysates reduced the H_2_O_2_ induced ROS and MDA levels in human intestinal Caco-2 cells

**Fig. 5:**
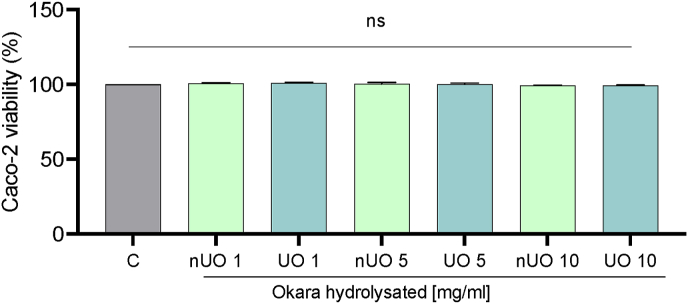
MTT assay of nUO and UO hydrolysates on Caco-2 cells. Bars represent the mean ± s.d. of three independent experiments performed in triplicate and statistically analyzed by One-way Anova followed by Tukey's post-hoc test. C: control, ns: not significant.

Considering the promising results obtained in the previous assays, experiments were performed for a deeper assessment of the antioxidant properties at cellular level. Notably, human intestinal Caco-2 cells have been chosen as cellular system of interest since being generally meant to be ingested, it is reasonable to consider that the intestinal barrier is the first physiological barrier that these hydrolysates encounter upon ingestion. MTT assay was performed to test the intestinal safety of okara hydrolysates, by excluding any potential cytotoxic effect. Results suggest that both untreated and ultrasonicated okara hydrolysates are safe for the intestinal cells at all the doses tested in the range 1.0–10.0 mg/mL, as shown in Fig. 5.

**Fig. 6:**
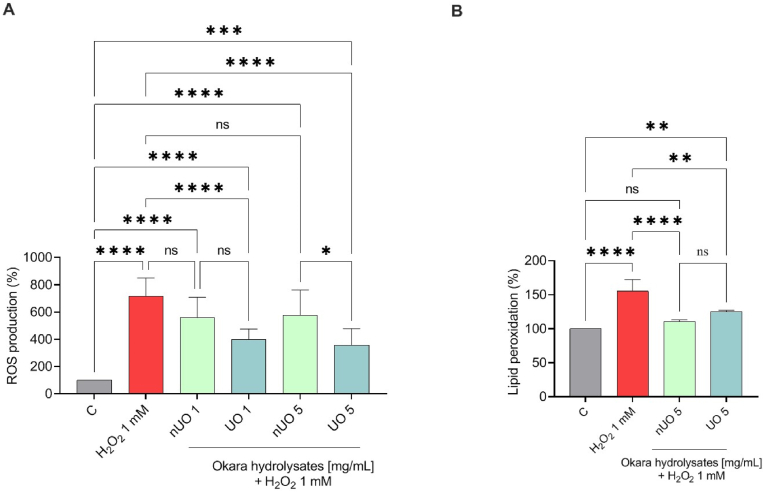
Modulation of intracellular H_2_O_2_-induced ROS levels (A) and lipid peroxidation (B) after the pretreatment with nUO and UO hydrolysates. Data represent the mean ± s.d. of three independent experiments performed in triplicate. All data sets were analyzed by One-way ANOVA followed by Tukey's post-hoc test. C: control sample. ns: not significant; (∗∗) *p* < 0.01, (∗∗∗) *p* < 0.001, (∗∗∗∗) *p* < 0.0001.

Hence, fluorometric intracellular ROS assay has been performed to evaluate nUO and UO effects in the ROS level modulation induced by H_2_O_2_ (1 mM). Results show that, after H_2_O_2_ treatment the ROS levels increase by 685.3 ± 121.1%. Pretreatments with both nUO and UO hydrolysates ameliorated H_2_O_2_-induced intracellular ROS level by 557.3 ± 150.9% and 397.3 ± 78.09% when tested at 1 mg/mL and by 602.4 ± 220.2% and 355.5 ± 122.7% at 5 mg/mL respectively, confirming their antioxidant potential (Fig. 6A). It is noticeable that, at the highest concentration, there is a significant difference between nUO and UO antioxidant activity, possibly due to a different composition of the mixture of peptides composing the ultrasound-treated sample. In addition, the capacity of nUO and UO hydrolysates to modulate the H_2_O_2_-induced lipid peroxidation in human intestinal Caco-2 cells was assessed by the MDA measurement. In accordance with the ROS results, after the H_2_O_2_ stress induction (1 mM), a significant increase of the lipid peroxidation was observed up to 155.2 ± 17.5% versus the control cells. Pretreatment with nUO and UO, both tested at the concentration of 5 mg/mL, resulted in a comparable decrease in lipid peroxidation levels by 110.9 ± 2.2% and 124.7 ± 2.2%, respectively (Fig. 6B).

### 3.5 Assessment of the intestinal trans-epithelial transport of soybean okara peptides using differentiated Caco-2 cells

**Fig. 7:**
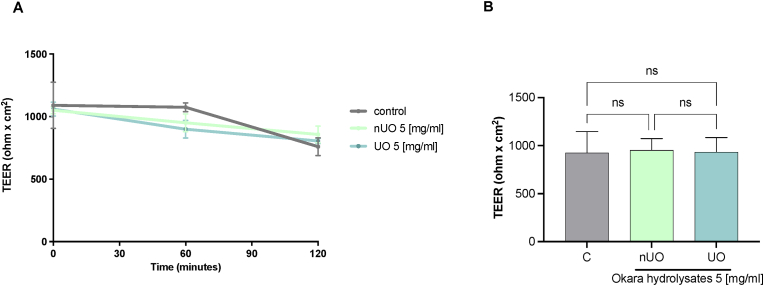
**TEER measurements in differentiated Caco-2 monolayer.** (A) Time course of TEER changes recorded in 2 h in untreated (control), nUO and UO hydrolysates-treated Caco-2 cells. (B) TEER values after 120 min. Data are the mean ± S.D. of an experiment performed in duplicate. ns: not significant.

To express biological activities, soybean okara hydrolysates must be absorbed at intestinal level. To study *in vitro* the trans-epithelial transport of soybean okara peptides, differentiated Caco-2 cells were employed. Caco-2 cells, when cultured in specific conditions, spontaneously acquire morphological and functional characteristics of mature enterocytes, expressing brush border peptidases and transporters, providing a valuable *in vitro* model for studying intestinal barrier. From a technical point of view, when differentiated on filters of Transwell system, these cells create a two-compartment system where the apical (AP) side mimics the intestinal lumen, while the basolateral (BL) side represents the intestinal vascular and lymphatic circulation in vivo (Bartolomei et al., 2024). Hence, this model assesses the ability of peptides to interact with the enterocytes and cross the intestinal epithelium, entering in the systemic circulation. For trans-epithelial transport experiments, nUO and UO (5 mg/mL) were incubated in the AP compartment. During the trans-epithelial transport experiments for evaluating the permeability and integrity of the Caco-2 cells polarized epithelial cell monolayer, TEER values were measured during the experiment. The results shown in Fig. 7 A and B indicate that both the both UO and nUO hydrolysates did not alter the intestinal monolayer permeability (no statistical significance different were observed in TEER values as a function of the time among samples).

Hence, AP and BL solutions were collected and analyzed by UHPLC-HRMS. Thus, focusing on dipeptides and tripeptides, results clearly indicated that out of the 316 and 315 nUO- and UO-derived peptides which were identified in the starting nUO and UO hydrolysates, only 34 and 41 peptides had been identified in the BL solution, respectively, clearly indicating that only 1.1% and 1.3% of the short peptides contained in nUO and UO are transported intact by differentiated intestinal cells. In addition, results suggest that out of the 34 transported nUO-derived peptides, 29 (85%) di- and 5 (15%) tri-peptides were identified, respectively. Similarly, out of the 41 transported UO-derived peptides, 35 (85%) and 6 (15%) are di- and tri-peptides, respectively.

**Fig. 8:**
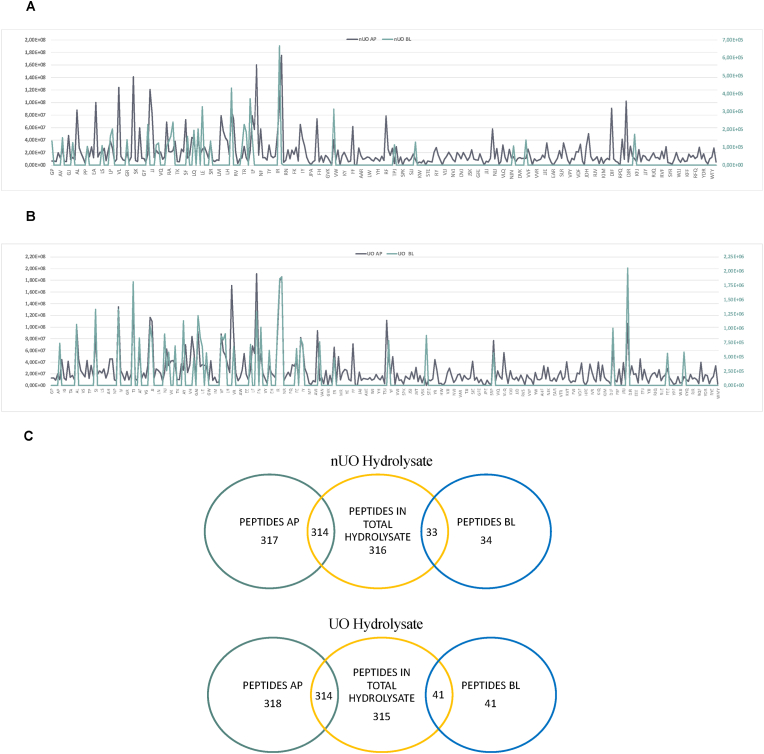
–Transport of nUO- and UO-derived peptides across differentiated Caco-2 cells. Distribution of short-sized peptides in AP and BL compartments for nUO (A) and UO (B) where on the x axis are shown the peptide sequences and on the y axis signal intensity. Schematic description of the peptides absorbed and stable for intestinal absorption (C)**.**

Moreover, our results indicate that out of the total 316 and 315 short peptides identified in the total nUO and UO hydrolysates, 317 (148 dipeptides and 169 tripeptides) and 318 (151 dipeptides and 167 tripeptides) are present in the nUO and UO AP solutions, respectively. Results indicated that peptides GP, EF, and SEJ originally present in the nUO sample, and GVK originally present in UO sample are completely metabolized by brush border, whereas peptides MR and EAR in the nUO and GP, PP, NP, and EF in the UO were generated by the intestinal peptidase activity (Fig. 8, Table S1).

To predict the absence of any toxic effect and identify the potential antioxidant peptides, the short peptides identified in both nUO and UO BL solutions were submitted to BIOPEP (https://biochemia.uwm.edu.pl/biopep-uwm/; accessed on May 23, 2024) and Toxinpred (http://crdd.osdd.net/raghava/toxinpred/; accessed on May 23, 2024) search. These tools are two open-access databases that allows to hypothesize potential biological activities of peptides, and the absence of toxic sequences based on the presence of some short amino acid sequences (Table 1). Among all the short peptides identified, 2 and 5 di-peptides with antioxidant activity were identified in nUO and UO BL solutions, respectively. 19 peptides with ACE-inhibitory activity were identified in both nUO and UO BL solutions while 15 and 12 peptides with DPPIV-inhibitory activity were found in nUO and UO BL solutions, respectively.

**Table 1:** – Potential peptide sequences identified in nUO and UO BL solutions with antioxidant activity, DPPIV and ACE-inhibitory activity, and non-toxicity prediction according to BIOPEP and Toxinpred databases.

BL solution	Peptide sequence	Signal intensity	Biological activity	Prediction
nUO	AR	1,07E+05	ACE inhibitor	non toxin
	RA	1,33E+05	DPPIV inhibitor	non toxin
	ER	3,13E+05	ACE inhibitor	non toxin
	EK	1,02E+05	DPPIV inhibitor, ACE inhibitor	non toxin
	GR	1,42E+05	ACE inhibitor	non toxin
	GP	1,35E+05	DPPIV inhibitor, ACE inhibitor	non toxin
	HP	1,61E+05	ACE inhibitor	non toxin
	IP	2,02E+05	DPPIV inhibitor, ACE inhibitor	non toxin
	LR	6,66E+05	ACE inhibitor	non toxin
	LN	1,24E+05	DPPIV inhibitor, ACE inhibitor	non toxin
	LQ	1,96E+05	ACE inhibitor	non toxin
	LK	2,02E+05	antioxidant	non toxin
	LF	1,59E+05	ACE inhibitor	non toxin
	LP	1,62E+05	ACE inhibitor	non toxin
	KP	2,28E+05	antioxidant, ACE inhibitor	non toxin
	PR	4,30E+05	ACE inhibitor	non toxin
	SP	1,26E+05	DPPIV inhibitor	non toxin
	TR	2,27E+05	DPPIV inhibitor	non toxin
	TDE	1,41E+05	DPPIV inhibitor	non toxin
	TPJ	1,14E+05	DPPIV inhibitor, ACE inhibitor	non toxin
	YP	3,71E+05	DPPIV inhibitor, ACE inhibitor	non toxin
	VA	1,55E+05	DPPIV inhibitor	non toxin
	VE	2,41E+05	DPPIV inhibitor, ACE inhibitor	non toxin
	VK	1,62E+05	DPPIV inhibitor, ACE inhibitor	non toxin
	VP	1,05E+05	DPPIV inhibitor, ACE inhibitor	non toxin
	JVT	1,28E+05	DPPIV inhibitor	non toxin
UO	AR	2,58E+05	ACE inhibitor, ACE inhibitor	non toxin
	AL	1,07E+06	DPPIV inhibitor	non toxin
	AF	8,29E+05	DPPIV inhibitor, ACE inhibitor	non toxin
	APF	8,77E+05	DPPIV inhibitor, ACE inhibitor	non toxin
	AY	1,14E+06	antioxidant, ACE inhibitor	non toxin
	AV	7,37E+05	DPPIV inhibitor, ACE inhibitor	non toxin
	RA	5,85E+05	DPPIV inhibitor	non toxin
	NF	1,02E+06	DPPIV inhibitor, ACE inhibitor	non toxin
	ER	4,82E+05	ACE inhibitor	non toxin
	ERE	5,88E+05	ACE inhibitor	non toxin
	IR	1,15E+06	antioxidant, ACE inhibitor	non toxin
	IE	5,73E+05	ACE inhibitor	non toxin
	IY	7,18E+05	antioxidant, ACE inhibitor	non toxin
	LR	1,87E+06	ACE inhibitor	non toxin
	LQ	4,82E+05	ACE inhibitor	non toxin
	LK	1,22E+06	antioxidant	non toxin
	FR	7,86E+05	ACE inhibitor	non toxin
	SY	9,05E+05	ACE inhibitor	non toxin
	TF	8,01E+05	DPPIV inhibitor, ACE inhibitor	non toxin
	TY	6,15E+05	antioxidant, DPPIV inhibitor	non toxin
	YP	7,20E+05	DPPIV inhibitor, ACE inhibitor	non toxin
	VR	6,85E+05	DPPIV inhibitor, ACE inhibitor	non toxin
	VL	1,33E+06	DPPIV inhibitor	non toxin
	VF	8,71E+05	DPPIV inhibitor, ACE inhibitor	non toxin

### 3.6 Evaluation of the hepatic safety profile at cellular level exploiting HepG2 cells: MTT experiment and transaminases release quantification with ELISAs assays

**Fig. 9:**
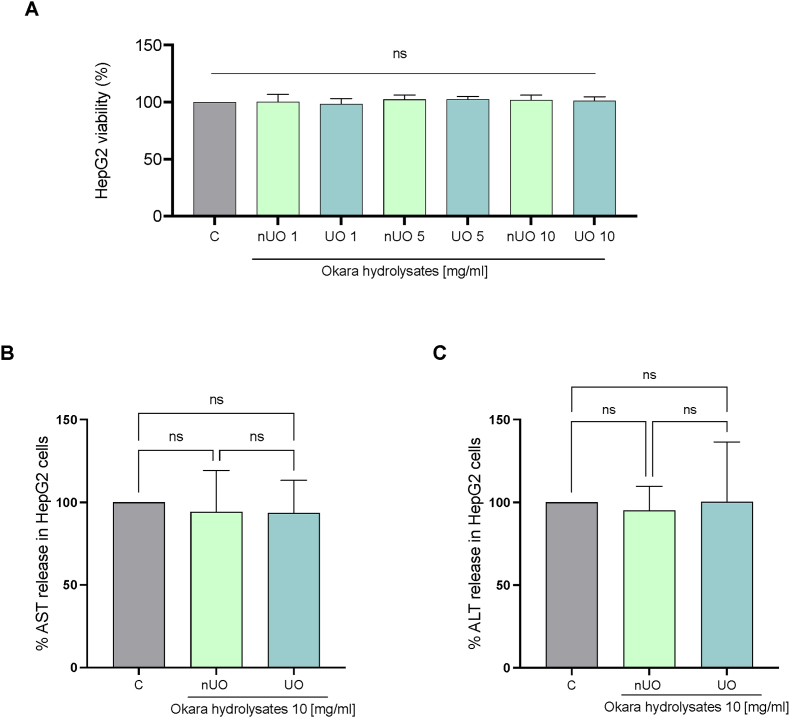
MTT assay of nUO and UO hydrolysates on HepG2 cells (A). Assessment of AST and ALT release in HepG2 cells supernatant after 24 h of treatment with nUO and UO (B–C). Bars represent the mean ± s.d. of two independent experiments performed in triplicate and statistically analyzed by One-way Anova followed by Tukey's post-hoc test. C: control, ns: not significant.

To verify and compare possible hepatotoxicity of nUO and UO hydrolysates, MTT experiments at different concentrations have been performed on HepG2 cells, demonstrating that there was no significant change in human hepatic cells viability treated with both soybean okara hydrolysates after 48 h up to 10 mg/mL (Fig. 9 A). Commonly recognized biomarkers for liver injury include aspartate aminotransferase (AST) and alanine aminotransferase (ALT) (Ji et al., 2010). Several authors have studied the hepatoprotective role of natural extracts on human liver cell lines, evaluating the leakage of AST and ALT enzymes from HepG2 cells (Niu et al., 2017; Pareek et al., 2013a, Pareek et al., 2013b). Hepatic transaminase release levels into the culture medium can be used to directly measure *in vitro* hepatotoxicity, being associated with cellular damage and injury, analyzing whether the natural compounds under investigation may exert some toxicity at cellular level.

After hepatic HepG2 cells were incubated for 24 h with soybean okara hydrolysates (nUO and UO) at high concentration (10 mg/mL), dedicated ELISA assays for the measurement of AST and ALT assays have been employed to evaluate whether 24 h treatment with nUO and UO hydrolysates could impair cellular integrity, increasing the levels of AST and ALT release in HepG2 cells supernatant. As reported in Fig. 9B and C there was no significant increase in the release of ALT and AST levels compared to control conditions, suggesting that these novel hydrolysates are not hepatotoxic at cellular level.

## 4 Discussion

Numerous studies have been conducted on the impact of ultrasonication on separation and extraction of food bioactive compounds; it is generally acknowledged that this method enhances the extraction of important components from soybeans, hence improving protein extraction overall (Rahman and Lamsal, 2021). Under normal circumstances, the intricacy of the soybean cellular microstructure affects the extraction of proteins; nevertheless, the cavitation phenomena make it possible to boost extraction yields from hexane-defatted soy flakes at the lab scale as previously described by Karki et al. (2009). Positive outcomes in terms of protein solubility and particle size reduction have also been reported for the enhancement of protein functionality from soybean protein isolate and concentrates (Tian et al., 2020). Furthermore, the solubility of isolated soy proteins in water is greatly increased by the ultrasound treatment (Jambrak et al., 2009). Therefore, it has previously been demonstrated (Aiello et al., 2021b) that high temperatures (60–80 °C) and ultrasound impact the chemical-physical properties and conformational changes of proteins extracted from soybean okara, enhancing the protein extraction yields and the proteins solubility without affecting the viscosity but modifying the protein secondary and tertiary structures. Based on this evidence, these protein structural modifications may also impact on the peptidomic profile obtained after the soybean okara protein enzymatic hydrolysis. In general, the enzymatic hydrolysis of food proteins is a powerful tool for obtaining peptide mixtures that beside their nutritional values, they exert also health promoting activities (Toldrá et al., 2018). Considering this peculiar issue, in this study we decided to combine ultrasonication and enzymatic hydrolysis for producing soybean okara hydrolysate, with the hypothesis that ultrasonication of the soybean okara coupled to enzymatic hydrolysis of the recovered proteins might lead to modulation of hydrolysate biological activity. Comparing the TPE of nUO and UO, it appears clear that ultrasonication led to an improvement of protein yield recovered from the soybean okara by-products and the combination of both alcalase and papain enzymes succeeded in co-digesting the total proteins over the time, reaching a % DH above than 50% (Fig. 1). Applying ultrafiltration method using a <3 KDa membrane cut-off, the nUO and UO fractions enriched in both medium and short-chain peptides have been obtained. In addition, results indicated that ultrasonication slightly but significantly increased the free a. a. contents in UO sample compared to the nUO samples (Fig. 2B). Results indicated that UO and nUO exert multifunctional activity, being DPP-IV and ACE inhibitors and antioxidant. Notably, the ultrasonication does not improve the *in vitro* DPP-IV and ACE activities, nevertheless these biological activities resulted efficiently and comparably reached for both samples. Particularly, the residual DPP-IV activity was almost halved at 2.5 mg/mL concentration for UO and nUO, and the ACE *in vitro* enzymatic inhibition was observed until 30% at 345 μg/mL, suggesting that both peptide mixtures are more active as ACE than DPP-IV inhibitors, respectively. These results are in line with literature evidence on soybean hydrolysates (Chatterjee et al., 2018) and, unlike them, our findings are the first highlighting the multifunctional ability of soybean okara peptides to inhibit both ACE and DPP-IV target. Interestingly, we have identified some di-peptides (VP, VA and YP) which are present as motives within the well characterized bioactive medium-chain peptides IAVPGEVA, IAVPTGVA and LPYP, endowed by ACE and DPP-IV inhibitory activity (Aiello et al., 2018; Lammi et al., 2015). The hydrolysis process generates peptides that can scavenge free radicals; these antioxidant peptides can donate hydrogen atoms or electrons to neutralize free radicals, preventing oxidative damage to lipids, proteins, and DNA. Following this mechanism, UO and nUO hydrolysates reduce oxidation through a combination of radical scavenging, ROS and lipid peroxidation reduction, respectively. UO samples exerted a direct scavenging antioxidant activity, but we did not observe an improved activity compared to the nUO samples (Fig. 4). On the contrary, at cellular levels in Caco-2 cells, UO is more active than nUO in reducing the H_2_O_2_ induced ROS (Fig. 4A), whereas both nUO and UO display a comparable ability to reduce the H_2_O_2_ induced lipid peroxidation in the same cellular system (Fig. 4B). These results are in line with literature suggesting that ultrasonication improves peptides antioxidant activities *in vitro* (Fadimu et al., 2022; Liu et al., 2019; Ningrum et al., 2023), and show that Okara hydrolysate, produced through ultrasonic pretreatment of the raw material and subsequent protein extraction followed by hydrolysis with papain and alcalase, exhibits antioxidant activity on Caco-2 intestinal cells. The biological activity of food derived components (such as food hydrolysates) is strictly correlated to its chemical composition. Since ultrasonication can enhance protein enzyme site exposure by inducing protein structural modifications and increasing surface area, thereby improving substrate accessibility for enzymatic hydrolysis (Qian et al., 2023), in this study, we evaluated the effect of ultrasonication coupled with enzymatic hydrolysis with food grade enzymes on the chemical composition of okara hydrolysate using UHPLC-HRMS analysis. The results (Table S1) show that while ultrasonication does not significantly generate new peptide species, its use as a pretreatment technique for okara proteins results in a notable modification of the relative abundance of specific peptides more than others. Regarding food bioactive peptides, their low intestinal bioavailability and low metabolic stability are two major obstacles that hinder their quick commercialization as nutraceuticals and/or functional foods (Amigo and Hernández-Ledesma, 2020). Short-chain peptides are more readily absorbed into the bloodstream, and it has been observed that the blood contains bioactive peptides following consumption of protein hydrolysates sourced from both plant and animal sources (Sato et al., 2008). In fact, the first physiological barrier that dietary peptides and/or hydrolysates meet is intestinal cells. Peptides are generally transported by the cells and partially metabolized by the same cellular environment when they encounter human enterocytes. However, it should be noted that the breakdown of bioactive peptides does not always result in the mixtures losing their bioactivity (Bollati et al., 2022; Lammi et al., 2021b). Therefore, the gut is a very complex physiological environment that directly affects the chemical compositions and peptidomics profiles of protein hydrolysates and/or peptides to actively control their bioactivity. Therefore, before conducting costly in vivo tests to prove the health-promoting effect of dietary protein hydrolysates, it is imperative to realize *in vitro* trials simulating the intestinal transport of these hydrolysates. Worldwide, the potential of food bioactive peptides to be transported by intestinal cells may be evaluated *in vitro* using differentiated human intestinal Caco-2 cells, which have been shown to be a trustworthy model. Food-derived peptides can often enter the bloodstream by one or more of the following pathways after crossing the intestinal brush-border membrane: (i) peptide transport 1 (PepT1)-mediated route, (ii) paracellular route via tight junctions (TJs), (iii) transcytosis route, and (iv) passive transcellular diffusion (Lammi et al., 2021c). Peptide size, charge, hydrophobicity, and peptidase-induced breakdown are undoubtedly some of the primary factors affecting absorption by one or more of these pathways. Due to PepT1's high capacity, low affinity, and high expression in the intestinal epithelium, short peptides like dipeptides and tripeptides are generally preferentially transported by it (Daniel, 2004). Conversely, highly hydrophobic peptides are transported either by transcytosis or simple passive transcellular diffusion. After the trans-epithelial transport experiments, peptides that crossed the cell monolayer were evaluated and analyzed, and the number of peptides that crossed the barrier represents their bioavailability.

However, peptides may be cleaved by brush-border peptidases that are responsible for breaking down larger peptides into smaller components that can be absorbed by the intestinal cells. Peptides that are resistant to degradation by these brush border enzymes have a higher chance of being absorbed intact into the bloodstream, where they can exert their physiological effects.

In this work, we identified 47% of dipeptides and 53% of tripeptides in both nUO and UO samples, respectively (Fig. 2). The cellular brush border activity dynamically modified the original peptidomic profile of each peptide mixtures. Indeed, results indicated that peptides GP, EF, and SEJ originally present in the nUO sample, and GVK originally present in UO sample are completely metabolized by brush border, whereas peptides MR and EAR in the nUO and GP, PP, NP, and EF in the UO were generated by the intestinal peptidase activity (Fig. 8, Table S1). Interestingly, we observed 34 and 41 peptides in nUO and UO BL solutions, which represents those peptides able to across differentiated intestinal cells. These results confirmed that Caco-2 cells act as a selective biological dynamic sieve, through which some peptides are enriched in the BL solution. In particular, the most abundant peptides found in nUO BL are ER, LE, YP, PR, LR and the most abundant peptides found in UO BL are TJ, LR, RJ, NJR (Table S1). These findings suggest that ultrasonication pretreatment may improve the peptides enrichment in BL solution (34 for nUO vs 41 for UO) besides increasing some peptides relative abundance. Furthermore, dipeptides and tripeptides are more easily absorbed and often show a better activity profile.

Finally, for assessing the nUO and UO safety profile, MTT experiments on intestinal Caco-2 and hepatic HepG2 cells and the quantification of AST and ALT in HepG2 cells supernatants, were performed, demonstrating that they are safe on cellular models useful to reproduce intestine and liver, which are the main organs involved in food and bioactive compounds absorption and metabolism. As a matter of fact, the safety of peptides in food applications is a concern due to the possibility of forming toxic or allergenic peptides from their parental proteins (Hartmann et al., 2007). Moreover, despite the advancements made with novel technologies, which are able to increase the extraction yields and biological activity of the bioactive peptides without altering the composition and structure of their hydrolysate compounds, they represent innovative techniques for the production of novel food-derived compounds, which would be included in the field of novel foods, for which the safety issues must be addressed before their approval in Europe (Hartmann et al., 2007; Peighambardoust et al., 2021). In conclusion, findings clearly indicated that both alcalase and papain are efficient in hydrolyzing soybean okara giving origin to protein hydrolysates enriched in short di- and tripeptides endowed by multifunctional activity and that can be easily transported by human intestinal cells. Hence, this work provides updated piece of information, which might have a relevant implication for an efficient and upcycled exploitation of soybean okara multifunctional peptides in health prevention.

## CRediT authorship contribution statement

**Lorenza d’Adduzio:** Investigation, Writing – review & editing. **Melissa Fanzaga:** Investigation. **Anna Laura Capriotti:** Supervision. **Enrico Taglioni:** Investigation. **Giovanna Boschin:** Investigation. **Aldo Laganà:** Writing – review & editing. **Lukas Rueller:** Investigation. **Josef Robert:** Writing – review & editing. **Antje van Gemmern:** Investigation. **Carlotta Bollati:** Investigation. **Carmen Lammi:** Conceptualization, Supervision, Writing – review & editing.

## Funding statement

This study was partially funded under the National Recovery and Resilience Plan (NRRP), Mission 4 Component 2 Investment 1.3 - Call for tender No. 341 of March 15, 2022 of Italian Ministry of University and Research funded by the 10.13039/501100000780European Union – NextGenerationEU; Project code PE00000003, Concession Decree No. 1550 of October 11, 2022 adopted by the Italian Ministry of University and Research, CUP D93C22000890001, Project title “**ON Foods - Research and innovation network on food and nutrition Sustainability, Safety and Security – Working ON Foods**”.

## Declaration of competing interest

Authors declare no conflict of interest.

## Data Availability

Data will be made available on request.
