# Supplementary material for: Ultrasonication coupled to enzymatic hydrolysis of soybean okara proteins for producing bioactive and bioavailable peptides
Source: Curr Res Food Sci. 2024 Nov 7;9:100919. doi: 10.1016/j.crfs.2024.100919 (PMC11582538; doi:10.1016/j.crfs.2024.100919)
Supplement: Multimedia component 1 [file mmc1.pdf]

Peptide	ID	Formula	RT [min]	Apicale 2h Okara	Apicale 2h Okara US
Gly-Pro	GP	C7 H12 N2 O3	1,26	6,82E+06	1,28E+07
Val-Gly	VG	C7 H14 N2 O3	1,719	6,78E+06	1,31E+07
Ala-Ser	AS	C6 H12 N2 O4	0,762	5,85E+06	9,39E+06
Ala-Pro	AP	C8 H14 N2 O3	1,745	1,97E+07	1,99E+07
Ala-Val	AV	C8 H16 N2 O3	2,49	1,15E+07	1,03E+07
Val-Ala	VA	C8 H16 N2 O3	1,627	2,73E+07	4,45E+07
Ile-Gly	IG	C8 H16 N2 O3	4,303	5,86E+06	1,27E+07
Leu-Gly	LG	C8 H16 N2 O3	4,654	5,89E+06	9,13E+06
Gly-Xle	GJ	C8 H16 N2 O3	6,236	4,72E+07	4,13E+07
Thr-Ala	TA	C7 H14 N2 O4	0,774	8,91E+06	1,38E+07
Ser-Pro	SP	C8 H14 N2 O4	1,247	1,26E+07	1,74E+07
Ala-Ile	AI	C9 H18 N2 O3	5,498	8,65E+06	9,67E+06
Ala-Leu	AL	C9 H18 N2 O3	6,143	8,78E+07	1,03E+08
Leu-Ala	LA	C9 H18 N2 O3	4,469	2,80E+07	4,51E+07
Ile-Ala	IA	C9 H18 N2 O3	4,016	1,85E+07	2,59E+07
Val-Ser	VS	C8 H16 N2 O4	1,048	1,07E+07	1,96E+07
Pro-Pro	PP	C10 H16 N2 O3	2,755	3,11E+06	1,51E+07
Val-Pro	VP	C10 H18 N2 O3	5,471	3,00E+07	4,27E+07
Thr-Pro	TP	C9 H16 N2 O4	2,24	1,37E+07	1,22E+07
Val-Val	VV	C10 H20 N2 O3	4,984	2,90E+07	3,43E+07
Glu-Ala	EA	C8 H14 N2 O5	0,865	1,20E+07	1,56E+07
Ser-Xle	SJ	C9 H18 N2 O4	5,758	1,00E+08	8,39E+07
Val-Thr	VT	C9 H18 N2 O4	1,244	1,32E+07	1,91E+07
Ile-Ser	IS	C9 H18 N2 O4	2,194	2,05E+07	2,49E+07
Leu-Ser	LS	C9 H18 N2 O4	2,454	2,00E+07	2,15E+07
Gly-Phe	GF	C11 H14 N2 O3	7,725	2,71E+07	2,84E+07
Phe-Gly	FG	C11 H14 N2 O3	6,547	7,06E+06	1,28E+07
Ala-His	AH	C9 H14 N4 O3	0,858	2,31E+07	2,29E+07
Leu-Pro	LP	C11 H20 N2 O3	8,117	3,82E+07	4,57E+07
Ile-Pro	IP	C11 H20 N2 O3	7,548	2,69E+07	4,55E+07
Asn-Pro	NP	C9 H15 N3 O4	1,31	5,39E+06	9,78E+06
Leu-Val	LV	C11 H22 N2 O3	7,711	9,15E+06	8,19E+06
Val-Leu	VL	C11 H22 N2 O3	8,337	1,24E+08	1,34E+08
Ile-Val	IV	C11 H22 N2 O3	7,039	1,44E+07	2,57E+07
Val-Ile	VI	C11 H22 N2 O3	7,516	8,28E+06	1,85E+07
Val-Asn	VN	C9 H17 N3 O4	0,952	7,29E+06	9,42E+06
Gly-Arg	GR	C8 H17 N5 O3	0,869	2,05E+07	2,40E+07
Val-Asp	VD	C9 H16 N2 O5	1,187	6,27E+06	9,65E+06
Xle-Thr	JT	C10 H20 N2 O4	2,728	1,30E+07	1,97E+07
Thr-Xle	TJ	C10 H20 N2 O4	6,37	1,41E+08	1,40E+08
Ser-Lys	SK	C9 H19 N3 O4	0,698	7,74E+06	1,21E+07
Ser-Glu	SE	C8 H14 N2 O6	0,767	6,16E+06	8,67E+06
Ala-Phe	AF	C12 H16 N2 O3	7,939	5,96E+07	5,95E+07
Phe-Ala	FA	C12 H16 N2 O3	6,529	1,10E+07	1,25E+07
Gly-Tyr	GY	C11 H14 N2 O4	4,854	1,07E+07	2,15E+07
Tyr-Gly	YG	C11 H14 N2 O4	4,111	5,30E+06	1,26E+07
Lys-Pro	KP	C11 H21 N3 O3	1,475	1,81E+07	3,37E+07
Xle-Xle	JJ	C12 H24 N2 O3	10,961	1,21E+08	1,17E+08
Xle-Xle	JJ	C12 H24 N2 O3	10,29	8,26E+07	1,09E+08

Xle-Xle	JJ	C12 H24 N2 O3	9,401	1,25E+07	1,32E+07
Ile-Asn	IN	C10 H19 N3 O4	1,764	2,35E+07	2,84E+07
Leu-Asn	LN	C10 H19 N3 O4	1,945	1,87E+07	2,58E+07
Val-Gln	VQ	C10 H19 N3 O4	1,215	2,10E+07	2,11E+07
Gly-Val-Ala	GVA	C10 H19 N3 O4	3,344	9,39E+06	7,45E+06
Asn-Xle	NJ	C10 H19 N3 O4	6,712	1,34E+07	2,26E+07
Ala-Arg	AR	C9 H19 N5 O3	0,913	6,89E+07	6,23E+07
Arg-Ala	RA	C9 H19 N5 O3	0,976	2,21E+07	3,48E+07
Val-Lys	VK	C11 H23 N3 O3	1,244	2,11E+07	4,19E+07
Val-Glu	VE	C10 H18 N2 O5	1,67	2,33E+07	4,30E+07
Asp-Xle	DJ	C10 H18 N2 O5	6,134	3,83E+07	3,66E+07
Thr-Lys	TK	C10 H21 N3 O4	0,805	5,76E+06	1,04E+07
Thr-Glu	TE	C9 H16 N2 O6	0,915	5,72E+06	1,01E+07
Thr-Met	TM	C9 H18 N2 O4 S	3,614	2,56E+07	3,73E+07
Ala-Tyr	AY	C12 H16 N2 O4	4,815	2,14E+07	2,63E+07
Ser-Phe	SF	C12 H16 N2 O4	7,362	7,27E+07	6,98E+07
His-Pro	HP	C11 H16 N4 O3	1,414	1,14E+07	2,22E+07
Val-His	VH	C11 H18 N4 O3	1,256	1,58E+07	3,52E+07
Ile-Gln	IQ	C11 H21 N3 O4	2,708	4,44E+07	8,40E+07
Leu-Gln	LQ	C11 H21 N3 O4	3,091	4,20E+07	5,83E+07
Gly-Ala-Xle	GAJ	C11 H21 N3 O4	7,07	1,29E+07	1,13E+07
Leu-Lys	LK	C12 H25 N3 O3	3,392	4,17E+07	9,20E+07
Ile-Lys	IK	C12 H25 N3 O3	2,85	1,75E+07	3,26E+07
Leu-Glu	LE	C11 H20 N2 O5	4,432	2,51E+07	3,59E+07
Glu-Xle	EJ	C11 H20 N2 O5	6,561	2,91E+07	3,45E+07
Ile-Glu	IE	C11 H20 N2 O5	3,826	2,01E+07	3,37E+07
Gly-Trp	GW	C13 H15 N3 O3	9,481	1,12E+07	9,39E+06
Ser-Arg	SR	C9 H19 N5 O4	0,715	3,72E+07	4,22E+07
Pro-Phe	PF	C14 H18 N2 O3	8,878	7,56E+06	8,97E+06
Ile-Met	IM	C11 H22 N2 O3 S	8,094	6,03E+06	1,45E+07
Met-Xle	MJ	C11 H22 N2 O3 S	9,66	8,42E+06	5,14E+06
Leu-Met	LM	C11 H22 N2 O3 S	8,656	7,57E+06	6,27E+06
Val-Phe	VF	C14 H20 N2 O3	9,941	7,91E+07	8,82E+07
Thr-Phe	TF	C13 H18 N2 O4	8,002	5,50E+07	5,82E+07
Ser-Tyr	SY	C12 H16 N2 O5	4,524	4,40E+07	5,21E+07
Leu-His	LH	C12 H20 N4 O3	6,019	3,89E+07	3,75E+07
Ile-His	IH	C12 H20 N4 O3	2,798	1,69E+07	2,25E+07
Pro-Arg	PR	C11 H21 N5 O3	2,59	8,68E+07	1,71E+08
Val-Arg	VR	C11 H23 N5 O3	1,953	7,44E+07	8,46E+07
Arg-Val	RV	C11 H23 N5 O3	3,552	2,09E+07	2,17E+07
Gln-Lys	QK	C11 H22 N4 O4	0,777	2,94E+06	8,18E+06
Ala-Trp	AW	C14 H17 N3 O3	9,532	1,02E+07	1,40E+07
Glu-Lys	EK	C11 H21 N3 O5	0,886	1,15E+07	2,68E+07
Thr-Arg	TR	C10 H21 N5 O4	0,743	2,99E+07	5,48E+07
Glu-Glu	EE	C10 H16 N2 O7	0,948	1,20E+07	1,90E+07
Met-Gln	MQ	C10 H19 N3 O4 S	1,765	5,44E+06	9,85E+06
Tyr-Pro	YP	C14 H18 N2 O4	7,064	2,23E+07	2,77E+07
Leu-Phe	LF	C15 H22 N2 O3	12,681	7,92E+07	6,77E+07
Ile-Phe	IF	C15 H22 N2 O3	11,904	5,69E+07	5,51E+07
Phe-Xle	FJ	C15 H22 N2 O3	12,502	1,60E+08	1,91E+08

Phe-Asn	FN	C13 H17 N3 O4	4,333	1,63E+07	2,49E+07
Asn-Phe	NF	C13 H17 N3 O4	7,54	5,78E+07	5,42E+07
Asp-Phe	DF	C13 H16 N2 O5	7,736	1,17E+07	2,06E+07
Val-Tyr	VY	C14 H20 N2 O4	6,457	1,23E+07	1,24E+07
Tyr-Val	YV	C14 H20 N2 O4	6,608	8,18E+06	3,76E+06
Thr-Tyr	TY	C13 H18 N2 O5	5,037	3,23E+07	3,31E+07
Glu-His	EH	C11 H16 N4 O5	0,881	1,49E+07	2,13E+07
Gly-Pro-Xle	GPJ	C13 H23 N3 O4	8,504	1,19E+07	1,75E+07
Val-Val-Ala	VVA	C13 H25 N3 O4	5,641	1,46E+07	1,36E+07
Ile-Arg	IR	C12 H25 N5 O3	4,348	4,78E+07	1,00E+08
Leu-Arg	LR	C12 H25 N5 O3	4,614	9,41E+07	1,79E+08
Arg-Xle	RJ	C12 H25 N5 O3	6,798	1,75E+08	1,86E+08
Asn-Arg	NR	C10 H20 N6 O4	0,741	4,67E+06	8,36E+06
Arg-Asn	RN	C10 H20 N6 O4	0,865	8,00E+06	1,67E+07
Ser-Trp	SW	C14 H17 N3 O4	9,085	2,44E+07	2,27E+07
Phe-Gln	FQ	C14 H19 N3 O4	5,252	1,01E+07	1,61E+07
Lys-Phe	KF	C15 H23 N3 O3	7,64	2,40E+07	3,60E+07
Phe-Lys	FK	C15 H23 N3 O3	5,364	1,73E+07	3,34E+07
Phe-Glu	FE	C14 H18 N2 O5	6,156	2,87E+07	4,30E+07
Glu-Phe	EF	C14 H18 N2 O5	8,261	6,60E+06	7,85E+06
Tyr-Xle	YJ	C15 H22 N2 O4	9,691	6,49E+07	8,20E+07
Ile-Tyr	IY	C15 H22 N2 O4	8,053	4,26E+07	6,48E+07
Leu-Tyr	LY	C15 H22 N2 O4	8,525	3,08E+07	2,97E+07
Asp-Tyr	DY	C13 H16 N2 O6	4,866	1,29E+07	1,91E+07
Met-Phe	MF	C14 H20 N2 O3 S	11,389	5,29E+06	4,14E+06
Xle-Pro-Ala	JPA	C14 H25 N3 O4	5,706	9,12E+05	1,85E+06
Gly-Xle-Xle	GJJ	C14 H27 N3 O4	12,335	5,28E+06	8,55E+06
Ala-Val-Xle	AVJ	C14 H27 N3 O4	10,253	7,45E+06	6,36E+06
His-Phe	HF	C15 H18 N4 O3	7,424	7,39E+07	9,39E+07
Phe-His	FH	C15 H18 N4 O3	5,195	5,48E+06	8,54E+06
Val-Ala-Asn	VAN	C12 H22 N4 O5	3,473	1,47E+07	1,41E+07
Gln-Arg	QR	C11 H22 N6 O4	1,004	4,72E+06	2,92E+06
Arg-Gln	RQ	C11 H22 N6 O4	0,885	1,57E+07	2,57E+07
Gly-Val-Lys	GVK	C13 H26 N4 O4	1,949	1,27E+07	-
Arg-Lys	RK	C12 H26 N6 O3	0,91	6,83E+06	1,75E+07
Gly-Pro-Me	GPM	C12 H21 N3 O4 S	6,157	8,94E+05	4,18E+06
Glu-Arg	ER	C11 H21 N5 O5	1,039	4,04E+07	6,56E+07
Val-Trp	VW	C16 H21 N3 O3	10,979	8,16E+06	5,49E+06
Thr-Trp	TW	C15 H19 N3 O4	9,509	2,42E+07	4,95E+07
Met-Arg	MR	C11 H23 N5 O3 S	2,94	-	9,46E+06
Tyr-Gln	YQ	C14 H19 N3 O5	2,645	1,52E+07	2,19E+07
Lys-Tyr	KY	C15 H23 N3 O4	5,02	2,08E+07	2,77E+07
Tyr-Glu	YE	C14 H18 N2 O6	3,783	1,30E+07	2,56E+07
Arg-His	RH	C12 H21 N7 O3	0,954	5,68E+06	1,06E+07
Met-Tyr	MY	C14 H20 N2 O4 S	7,643	6,94E+06	7,98E+06
Phe-Phe	FF	C18 H20 N2 O3	13,52	6,18E+07	7,12E+07
Ala-Pro-Glu	APE	C13 H21 N3 O6	2,248	3,65E+05	5,18E+05
Xle-Pro-Ser	JPS	C14 H25 N3 O5	6,691	5,22E+05	4,90E+05
Xle-Ala-Xle	JAJ	C15 H29 N3 O4	12,22	2,74E+07	2,28E+07
Ala-Ala-Arg	AAR	C12 H24 N6 O4	1,089	1,54E+07	8,61E+06

Ala-Val-Lys	AVK	C14 H28 N4 O4	1,841	9,36E+06	1,24E+07
Ala-Val-Glu	AVE	C13 H23 N3 O6	3,255	1,07E+07	1,13E+07
Ile-Trp	IW	C17 H23 N3 O3	12,445	1,31E+07	1,06E+07
Leu-Trp	LW	C17 H23 N3 O3	13,099	1,23E+07	1,35E+07
Trp-Xle	WJ	C17 H23 N3 O3	14,035	8,68E+06	9,20E+06
Ser-Val-Ile	SVI	C14 H27 N3 O5	9,742	6,89E+06	1,12E+07
Ser-Val-Leu	SVL	C14 H27 N3 O5	10,125	1,17E+07	1,88E+07
Tyr-His	YH	C15 H18 N4 O4	4,823	9,65E+06	1,29E+07
Ser-Val-Asp	SVD	C12 H21 N3 O7	2,107	6,55E+06	9,79E+06
Thr-Xle-Ser	TJS	C13 H25 N3 O6	4,37	1,43E+07	1,68E+07
Thr-Ser-Xle	TSJ	C13 H25 N3 O6	7,681	7,50E+06	3,03E+06
Arg-Phe	RF	C15 H23 N5 O3	8,158	7,84E+07	1,12E+08
Phe-Arg	FR	C15 H23 N5 O3	6,216	2,49E+07	4,64E+07
Tyr-Phe	YF	C18 H20 N2 O4	11,031	1,57E+07	2,08E+07
Phe-Tyr	FY	C18 H20 N2 O4	9,911	2,69E+07	4,94E+07
Thr-Pro-Xle	TPJ	C15 H27 N3 O5	8,82	2,64E+06	1,82E+06
Val-Val-Xle	VVJ	C16 H31 N3 O4	11,663	2,97E+07	2,09E+07
Ala-Xle-Gln	AJQ	C14 H26 N4 O5	4,888	3,59E+06	8,86E+06
Val-Val-Asn	VVN	C14 H26 N4 O5	2,744	1,32E+07	9,79E+06
Ser-Pro-Lys	SPK	C14 H26 N4 O5	1,183	6,68E+05	2,66E+06
Ala-Xle-Lys	AJK	C15 H30 N4 O4	4,408	8,96E+06	1,64E+07
Ala-Xle-Glu	AJE	C14 H25 N3 O6	5,675	1,16E+07	1,31E+07
Xle-Ser-Xle	JSJ	C15 H29 N3 O5	12,504	6,67E+06	1,05E+07
Ser-Xle-Xle	SJJ	C15 H29 N3 O5	12,16	1,04E+07	2,31E+07
Ser-Xle-Xle	SJJ	C15 H29 N3 O5	12,749	1,79E+07	1,13E+07
Xle-Val-Thr	JVT	C15 H29 N3 O5	7,273	9,12E+06	3,10E+07
Trp-Gln	WQ	C16 H20 N4 O4	6,814	3,05E+06	6,50E+06
Lys-Trp	KW	C17 H24 N4 O3	9,139	4,92E+06	1,71E+07
Val-Ser-Lys	VSK	C14 H28 N4 O5	1,778	3,83E+06	4,78E+06
Glu-Trp	EW	C16 H19 N3 O5	9,773	7,96E+06	1,58E+07
Ala-Pro-Phe	APF	C17 H23 N3 O4	10,579	7,13E+05	2,59E+05
Ser-Thr-Glu	STE	C12 H21 N3 O8	1,003	6,85E+06	9,45E+06
Gly-Phe-Xle	GFJ	C17 H25 N3 O4	14,034	6,00E+06	8,20E+06
Xle-Gly-Phe	JGF	C17 H25 N3 O4	13,732	5,30E+06	1,15E+07
Tyr-Arg	YR	C15 H23 N5 O4	4,16	2,11E+07	4,45E+07
Arg-Tyr	RY	C15 H23 N5 O4	5,456	2,81E+07	2,67E+07
Ala-Xle-His	AJH	C15 H25 N5 O4	4,457	1,66E+07	2,98E+06
His-Trp	HW	C17 H19 N5 O3	9,087	8,30E+06	3,93E+06
Xle-Pro-Asn	JPN	C15 H26 N4 O5	6,6	8,12E+05	6,06E+05
Val-Xle-Xle	VJJ	C17 H33 N3 O4	13,53	9,27E+06	6,05E+06
Val-Xle-Xle	VJJ	C17 H33 N3 O4	12,929	1,45E+07	2,17E+07
Tyr-Tyr	YY	C18 H20 N2 O5	7,771	2,06E+07	2,98E+07
Val-Val-Gln	VVQ	C15 H28 N4 O5	4,009	1,51E+07	1,79E+07
Val-Asn-Xle	NVJ	C15 H28 N4 O5	10,233	9,00E+06	5,40E+06
Gly-Leu-Arg	GLR	C14 H28 N6 O4	5,957	5,61E+06	9,46E+06
Gly-Ile-Arg	GIR	C14 H28 N6 O4	5,591	2,03E+07	1,83E+07
Val-Ala-Arg	VAR	C14 H28 N6 O4	3,234	1,67E+07	1,98E+07
Asp-Val-Xle	DVJ	C15 H27 N3 O6	10,376	1,00E+07	7,86E+06
Xle-Thr-Xle	JTJ	C16 H31 N3 O5	12,61	2,02E+07	1,76E+07
Thr-Xle-Xle	TJJ	C16 H31 N3 O5	11,939	1,42E+07	1,26E+07

Thr-Val-Lys	TVK	C15 H30 N4 O5	2,063	7,74E+06	1,04E+07
Xle-Ser-Lys	JSK	C15 H30 N4 O5	4,332	1,28E+07	1,32E+07
Ser-Xle-Glu	SJE	C14 H25 N3 O7	5,958	2,46E+07	4,14E+07
Asp-Thr-Xle	DTJ	C14 H25 N3 O7	7,449	9,65E+06	9,25E+06
Ser-Glu-Xle	SEJ	C14 H25 N3 O7	7,558	8,37E+06	1,39E+07
Gly-Phe-Glu	GFE	C16 H21 N3 O6	7,559	8,02E+06	4,98E+06
Val-Ser-Phe	VSF	C17 H25 N3 O5	11,475	1,81E+07	1,05E+07
Ser-Xle-His	SJH	C15 H25 N5 O5	4,621	8,80E+06	2,87E+06
Xle-Pro-Glu	JPE	C16 H27 N3 O6	7,853	4,66E+05	8,84E+05
Xle-Xle-Xle	JJJ	C18 H35 N3 O4	14,255	6,47E+06	9,15E+06
Xle-Xle-Xle	JJJ	C18 H35 N3 O4	15,647	5,16E+06	3,63E+06
Ser-Arg-Pro	SRP	C14 H26 N6 O5	2,066	1,09E+07	1,44E+06
Asn-Xle-Xle	NJJ	C16 H30 N4 O5	12,081	5,78E+07	7,70E+07
Asn-Xle-Xle	NJJ	C16 H30 N4 O5	12,641	1,01E+07	7,90E+06
Val-Ile-Gln	VIQ	C16 H30 N4 O5	5,804	2,66E+07	2,48E+07
Xle-Val-Gln	JVQ	C16 H30 N4 O5	5,689	1,74E+06	1,56E+07
Gln-Val-Xle	QVJ	C16 H30 N4 O5	10,057	5,94E+06	1,10E+07
Val-Leu-Gln	VLQ	C15 H30 N6 O4	5,886	3,20E+07	5,63E+07
Xle-Ala-Arg	JAR	C15 H30 N6 O4	5,515	1,25E+07	2,18E+07
Arg-Ala-Xle	RAJ	C15 H30 N6 O4	7,736	5,55E+06	3,59E+06
Lys-Val-Xle	KVJ	C17 H34 N4 O4	9,333	2,52E+07	2,55E+07
Asn-Xle-Asn	NJN	C14 H25 N5 O6	3,711	1,15E+07	1,21E+07
Val-Xle-Glu	VJE	C16 H29 N3 O6	6,888	1,36E+07	1,02E+07
Xle-Xle-Asp	JJD	C16 H29 N3 O6	8,059	5,59E+06	1,44E+07
Glu-Val-Xle	EVJ	C16 H29 N3 O6	10,032	1,04E+07	7,85E+06
Asp-Val-Lys	DVK	C15 H28 N4 O6	1,818	1,23E+07	1,79E+07
Arg-Val-Ser	RVS	C14 H28 N6 O5	2,324	1,08E+07	1,10E+07
Val-Pro-Phe	VPF	C19 H27 N3 O4	12,436	1,08E+07	1,32E+06
Thr-Asp-Glu	TDE	C13 H21 N3 O9	1,116	1,10E+07	1,83E+07
Val-Val-Phe	VVF	C19 H29 N3 O4	12,981	2,60E+07	1,30E+07
Phe-Xle-Ser	FJS	C18 H27 N3 O5	10,082	1,46E+07	1,01E+07
Xle-Pro-His	JPH	C17 H27 N5 O4	7,693	7,01E+05	1,83E+06
Tyr-Trp	YW	C20 H21 N3 O4	11,879	1,02E+07	2,24E+07
Val-Val-Arg	VVR	C16 H32 N6 O4	4,976	6,92E+06	1,05E+07
Lys-Xle-Xle	KJJ	C18 H36 N4 O4	11,803	8,21E+06	9,51E+06
Ala-His-Phe	AHF	C18 H23 N5 O4	8,974	9,94E+06	1,22E+07
Asn-Xle-Gln	NJQ	C15 H27 N5 O6	4,758	1,59E+07	3,36E+07
Xle-Xle-Glu	JJE	C17 H31 N3 O6	8,779	1,14E+07	1,13E+07
Asn-Xle-Lys	NJK	C16 H31 N5 O5	4,646	3,54E+07	3,10E+07
Lys-Xle-Asn	KJN	C16 H31 N5 O5	3,405	1,32E+07	8,68E+06
Val-Gln-Glu	VQE	C15 H26 N4 O7	4,007	5,07E+06	8,02E+06
Glu-Ala-Arg	EAR	C14 H26 N6 O6	2,414	-	1,32E+07
Val-Ala-Trp	VAW	C19 H26 N4 O4	12,457	4,94E+06	7,17E+06
Lys-Val-Glu	KVE	C16 H30 N4 O6	2,785	1,08E+07	1,77E+07
Val-Thr-Arg	VTR	C15 H30 N6 O5	3,3	2,35E+07	2,48E+07
Ser-Leu-Arg	SLR	C15 H30 N6 O5	5,826	1,08E+07	1,01E+07
Ser-Ile-Arg	SIR	C15 H30 N6 O5	5,464	3,54E+07	1,45E+07
Arg-Val-Thr	RVT	C15 H30 N6 O5	4,041	2,30E+07	4,07E+07
Asp-Xle-Glu	DJE	C15 H25 N3 O8	5,651	8,11E+06	1,03E+07
Val-Pro-Tyr	VPY	C19 H27 N3 O5	9,294	2,23E+06	4,72E+06

Phe-Val-Xle	FVJ	C20 H31 N3 O4	15,526	1,11E+07	8,09E+06
Val-Xle-Phe	VJF	C20 H31 N3 O4	15,071	8,43E+06	6,44E+06
Xle-Val-Phe	JVF	C20 H31 N3 O4	14,413	2,15E+07	2,13E+07
Val-Asp-Phe	VDF	C18 H25 N3 O6	11,821	2,12E+07	1,97E+07
Val-Phe-Asp	VFD	C18 H25 N3 O6	8,405	3,34E+07	3,87E+07
Phe-Thr-Xle	FTJ	C19 H29 N3 O5	14,526	6,46E+06	6,40E+06
His-Pro-Glu	HPE	C16 H23 N5 O6	3,839	5,03E+05	4,54E+05
Xle-Asp-His	JDH	C16 H25 N5 O6	7,358	3,14E+07	7,70E+06
Arg-Val-Xle	RVJ	C17 H34 N6 O4	9,748	5,01E+07	3,68E+07
Ile-Val-Arg	IVR	C17 H34 N6 O4	6,304	1,41E+07	2,29E+07
Leu-Val-Arg	LVR	C17 H34 N6 O4	6,496	7,38E+06	1,14E+07
Arg-Xle-Val	RJV	C17 H34 N6 O4	9,22	9,83E+06	6,15E+06
Lys-Ile-Gln	KIQ	C17 H33 N5 O5	4,549	1,34E+07	4,03E+07
Lys-Leu-Gln	KLQ	C17 H33 N5 O5	4,941	2,67E+06	8,51E+06
Glu-Xle-Glu	EJE	C16 H27 N3 O8	6,229	1,21E+07	3,50E+07
Lys-Xle-Met	KJM	C17 H34 N4 O4 S	9,129	3,03E+06	1,45E+07
Xle-Pro-Tyr	JPY	C20 H29 N3 O5	10,463	7,87E+06	9,26E+06
Xle-Xle-Phe	JJF	C21 H33 N3 O4	15,587	1,10E+07	6,98E+06
Asp-Leu-Phe	DLF	C19 H27 N3 O6	14,489	8,42E+06	1,70E+06
Asp-Ile-Phe	DIF	C19 H27 N3 O6	13,826	9,07E+07	6,59E+07
Xle-Val-Tyr	JVY	C20 H31 N3 O5	10,661	1,02E+07	9,03E+06
Phe-Ser-Phe	FSF	C21 H25 N3 O5	14,958	6,19E+06	6,04E+06
His-Xle-Met	HJM	C17 H29 N5 O4 S	9,545	4,76E+06	2,80E+07
Arg-Pro-Gln	RPQ	C16 H29 N7 O5	2,23	2,04E+05	2,23E+05
Xle-Arg-Xle	JRJ	C18 H36 N6 O4	11,451	4,13E+07	3,94E+07
Xle-Arg-Xle	JRJ	C18 H36 N6 O4	12,109	9,42E+06	7,74E+06
Asn-Xle-Arg	NJR	C16 H31 N7 O5	5,782	1,02E+08	1,06E+08
Asp-Xle-Arg	DJR	C16 H30 N6 O6	5,57	5,21E+06	1,73E+07
Glu-Val-Arg	EVR	C16 H30 N6 O6	3,518	3,06E+07	3,37E+07
Lys-Glu-Glu	KEE	C16 H28 N4 O8	1,116	1,40E+07	1,89E+07
Glu-Glu-Glu	EEE	C15 H23 N3 O10	1,467	1,04E+07	2,00E+07
Lys-Phe-Xle	KFJ	C21 H34 N4 O4	12,906	5,72E+06	4,54E+06
Phe-Xle-Lys	FJK	C21 H34 N4 O4	9,569	2,51E+07	4,56E+07
Glu-Phe-Xle	EFJ	C20 H29 N3 O6	13,775	5,48E+06	9,76E+06
Phe-Xle-Glu	FJE	C20 H29 N3 O6	10,772	1,55E+07	2,75E+07
Xle-Xle-Tyr	JJY	C21 H33 N3 O5	11,703	1,38E+07	1,22E+07
Tyr-Xle-Xle	YJJ	C21 H33 N3 O5	14,215	8,92E+06	3,99E+06
Phe-Asn-Glu	FNE	C18 H24 N4 O7	7,142	7,35E+06	1,50E+07
His-Phe-Xle	HFJ	C21 H29 N5 O4	13,273	1,37E+07	2,03E+07
Arg-Xle-Gln	RJQ	C17 H33 N7 O5	5,547	1,98E+07	2,21E+07
His-Phe-Asn	HFN	C19 H24 N6 O5	5,884	1,18E+07	1,40E+07
Arg-Ile-Glu	RIE	C17 H32 N6 O6	6,077	1,23E+07	2,73E+07
Arg-Leu-Glu	RLE	C17 H32 N6 O6	6,477	9,21E+06	8,23E+06
Arg-Val-Phe	RVF	C20 H32 N6 O4	10,918	2,25E+07	1,51E+07
Lys-Xle-Tyr	KJY	C21 H34 N4 O5	9,304	1,07E+07	1,78E+07
Phe-Glu-Glu	FEE	C19 H25 N3 O8	7,71	2,94E+07	2,90E+07
Glu-Xle-Tyr	EJY	C20 H29 N3 O7	9,636	4,18E+06	1,34E+07
Ser-Tyr-Arg	SYR	C18 H28 N6 O6	5,068	3,30E+06	7,82E+06
Tyr-Pro-Phe	YPF	C23 H27 N3 O5	13,425	1,82E+06	2,45E+06
Phe-Phe-Xle	FFJ	C24 H31 N3 O4	18,011	9,70E+06	3,57E+06

sn-Phe-Ph	NFF	C22 H26 N4 O5	14,882	2,04E+07	1,52E+07
Trp-Xle-Xle	WJJ	C23 H34 N4 O4	18,429	9,50E+06	4,40E+06
Trp-Xle-Xle	WJJ	C23 H34 N4 O4	17,99	4,90E+06	5,02E+06
Glu-Arg-Glu	ERE	C16 H28 N6 O8	1,698	1,12E+07	1,55E+07
Lys-Tyr-Gln	KYQ	C20 H31 N5 O6	4,114	6,17E+06	1,06E+07
ys-Phe-Phe	KFF	C24 H32 N4 O4	13,892	6,79E+06	6,93E+06
he-Phe-Glu	FFE	C23 H27 N3 O6	11,905	1,77E+07	1,86E+07
Arg-Ile-Arg	RIR	C18 H37 N9 O4	6,03	1,28E+07	1,16E+07
Arg-Leu-Arg	RLR	C18 H37 N9 O4	6,339	1,58E+07	1,31E+07
Arg-Phe-Gln	RFQ	C20 H31 N7 O5	6,931	1,08E+07	1,01E+07
Arg-Gln-Phe	RQF	C20 H31 N7 O5	8,454	1,36E+07	3,27E+06
Arg-Phe-Glu	RFE	C20 H30 N6 O6	7,714	2,89E+07	3,97E+07
Tyr-Xle-Arg	YJR	C21 H34 N6 O5	7,965	1,03E+07	6,99E+06
Tyr-Asp-Arg	YDR	C19 H28 N6 O7	6,494	1,84E+07	1,82E+07
Trp-Gln-Glu	WQE	C21 H27 N5 O7	8,503	6,24E+06	1,70E+07
Lys-Tyr-Arg	KYR	C21 H35 N7 O5	5,03	2,04E+06	3,09E+06
Arg-Tyr-Glu	RYE	C20 H30 N6 O7	5,634	1,04E+07	1,14E+07
Trp-Thr-Tyr	WTY	C24 H28 N4 O6	12,574	1,27E+07	1,52E+07
Arg-Phe-Tyr	RFY	C24 H32 N6 O5	10,996	2,73E+07	3,37E+07
rp-Met-Ty	WMY	C25 H30 N4 O5 S	14,359	5,02E+06	2,55E+06

Apicale T0 Okara	Apicale T0 Okara US	BL 2h Okara	BL 2h Okara US
-	-	1,35E+05	-
9,26E+06	1,24E+07	-	-
4,75E+06	4,96E+06	-	-
6,74E+05	7,85E+05	-	-
9,11E+06	7,66E+06	-	7,37E+05
2,39E+07	3,68E+07	1,55E+05	-
7,84E+06	9,71E+06	-	-
7,31E+06	8,05E+06	-	-
4,56E+07	3,15E+07	-	-
5,66E+06	1,33E+07	-	-
2,17E+05	2,50E+05	1,26E+05	-
9,54E+06	9,54E+06	-	-
9,49E+07	9,28E+07	-	1,07E+06
3,51E+07	4,33E+07	-	-
2,35E+07	1,78E+07	-	-
1,30E+07	1,81E+07	-	-
8,45E+04	-	-	-
6,27E+05	8,98E+05	1,05E+05	-
1,82E+05	1,79E+05	-	-
3,70E+07	3,04E+07	-	-
6,88E+06	8,55E+06	-	-
9,48E+07	6,98E+07	-	1,33E+06
1,61E+07	1,46E+07	-	-
1,80E+07	1,55E+07	-	-
1,88E+07	1,54E+07	1,10E+05	-
2,82E+07	2,42E+07	-	-
5,79E+06	8,85E+06	-	-
2,52E+06	3,44E+06	-	-
6,27E+05	8,49E+05	1,62E+05	-
3,12E+05	4,01E+05	2,02E+05	-
4,18E+05	-	-	-
1,11E+07	7,97E+06	-	-
1,46E+08	1,26E+08	-	1,33E+06
1,49E+07	1,97E+07	-	-
9,57E+06	1,53E+07	-	-
8,98E+06	1,64E+07	-	-
2,38E+07	2,04E+07	1,42E+05	-
4,81E+06	5,94E+06	-	-
1,19E+07	1,18E+07	-	-
1,43E+08	1,20E+08	-	1,81E+06
9,47E+06	1,01E+07	-	-
7,86E+06	8,96E+06	-	-
6,73E+07	5,95E+07	-	8,29E+05
7,48E+06	8,06E+06	-	-
1,18E+07	1,28E+07	-	-
3,06E+06	7,67E+06	-	-
1,71E+05	2,08E+05	2,28E+05	-
1,67E+08	1,51E+08	-	1,04E+06
8,73E+07	8,96E+07	-	8,19E+05

1,37E+07	9,85E+06	-	-
2,32E+07	2,14E+07	1,13E+05	-
1,92E+07	2,52E+07	1,24E+05	-
2,57E+07	1,63E+07	-	-
8,21E+06	4,06E+06	-	-
8,36E+06	1,43E+07	-	9,02E+05
5,37E+07	5,26E+07	1,07E+05	2,58E+05
1,48E+07	1,52E+07	1,33E+05	5,85E+05
1,67E+07	2,04E+07	1,62E+05	-
2,13E+07	3,33E+07	2,41E+05	-
3,49E+07	1,84E+07	-	6,91E+05
1,76E+06	2,63E+06	-	-
3,56E+06	2,88E+06	-	-
1,70E+07	1,68E+07	-	-
1,24E+07	9,94E+06	-	1,14E+06
7,23E+07	6,00E+07	-	-
6,57E+05	1,20E+06	1,61E+05	-
1,02E+07	1,63E+07	-	-
4,80E+07	6,64E+07	-	-
4,77E+07	4,21E+07	1,96E+05	4,82E+05
1,47E+07	9,89E+06	-	-
5,27E+07	7,57E+07	2,02E+05	1,22E+06
1,94E+07	2,54E+07	-	8,39E+05
1,91E+07	2,21E+07	3,27E+05	6,80E+05
2,61E+07	1,95E+07	-	-
1,80E+07	1,75E+07	-	5,73E+05
1,09E+07	7,61E+06	-	-
2,51E+07	2,59E+07	1,35E+05	-
3,25E+06	3,19E+06	-	-
7,44E+06	1,19E+07	-	-
1,64E+07	1,23E+07	-	-
9,04E+06	8,10E+06	-	-
1,06E+08	9,67E+07	-	8,71E+05
6,24E+07	5,20E+07	-	8,01E+05
3,79E+07	3,15E+07	-	9,05E+05
3,48E+07	2,73E+07	-	-
2,43E+07	2,30E+07	-	-
2,65E+06	3,60E+06	4,30E+05	-
7,28E+07	8,91E+07	-	6,85E+05
3,66E+06	3,59E+06	-	-
7,12E+05	9,56E+05	-	-
1,16E+07	1,45E+07	-	-
1,39E+07	1,80E+07	1,02E+05	-
1,55E+07	2,29E+07	2,27E+05	-
8,72E+06	1,18E+07	1,86E+05	-
5,17E+06	8,74E+06	-	-
1,56E+06	1,22E+06	3,71E+05	7,20E+05
1,16E+08	9,89E+07	1,59E+05	-
6,04E+07	4,86E+07	-	-
1,96E+08	1,92E+08	-	1,29E+06

1,56E+07	1,72E+07	-	-
5,72E+07	4,52E+07	-	1,02E+06
8,55E+06	1,50E+07	-	-
1,38E+07	1,14E+07	-	-
3,67E+06	3,39E+06	-	-
3,49E+07	2,93E+07	-	6,15E+05
7,24E+06	8,31E+06	-	-
2,31E+07	2,30E+07	-	-
1,62E+07	1,13E+07	-	-
5,30E+07	7,42E+07	-	1,15E+06
1,28E+08	1,72E+08	6,66E+05	1,87E+06
2,20E+08	1,87E+08	9,72E+03	1,90E+06
7,50E+06	1,14E+07	-	-
4,30E+05	7,72E+05	-	-
2,48E+07	2,12E+07	-	-
9,90E+06	9,33E+06	-	-
3,39E+07	3,32E+07	-	-
2,49E+07	2,92E+07	-	-
2,04E+07	2,30E+07	-	6,49E+05
-	-	-	-
6,89E+07	7,11E+07	-	8,19E+05
4,48E+07	5,72E+07	-	7,18E+05
3,70E+07	3,38E+07	-	-
8,15E+06	9,77E+06	-	-
8,63E+06	8,03E+06	-	-
8,16E+06	4,12E+06	-	-
5,65E+06	7,77E+06	-	-
8,70E+06	6,30E+06	-	-
6,81E+07	7,18E+07	-	-
4,41E+06	6,03E+06	-	7,63E+05
1,37E+07	9,97E+06	-	-
6,25E+06	5,51E+06	-	-
1,18E+07	1,15E+07	-	-
1,71E+06	2,17E+05	-	-
6,66E+06	8,66E+06	-	-
3,39E+06	1,23E+07	-	-
2,98E+07	4,25E+07	3,13E+05	4,82E+05
4,29E+04	4,39E+04	-	-
2,79E+07	4,97E+07	-	-
7,57E+06	9,35E+06	-	-
1,26E+07	1,51E+07	-	-
2,67E+07	2,35E+07	-	-
9,27E+06	1,34E+07	-	-
1,59E+06	1,51E+06	-	-
9,40E+06	1,17E+07	-	-
7,04E+07	7,13E+07	-	-
4,03E+06	9,22E+06	-	-
9,70E+06	7,17E+06	-	-
3,14E+07	2,38E+07	-	-
1,10E+07	6,30E+06	-	-

9,98E+06	1,13E+07	-	-
1,85E+06	1,59E+06	-	-
1,55E+07	1,16E+07	-	-
1,66E+07	2,01E+07	-	-
1,03E+07	9,07E+06	-	-
6,56E+06	9,37E+06	-	-
1,16E+07	1,75E+07	-	-
7,86E+06	7,96E+06	-	-
3,97E+06	5,56E+06	-	-
1,42E+07	1,36E+07	-	-
8,62E+06	2,88E+06	-	-
7,88E+07	8,90E+07	-	-
2,35E+07	3,44E+07	-	7,86E+05
1,41E+07	1,61E+07	-	-
2,04E+07	3,48E+07	-	-
2,77E+07	1,64E+07	1,14E+05	-
3,97E+07	2,19E+07	-	-
3,82E+06	7,24E+06	-	-
1,33E+07	7,76E+06	-	-
5,94E+06	2,37E+07	-	-
1,66E+07	1,92E+07	-	-
1,04E+07	7,35E+06	-	-
1,24E+07	1,68E+07	-	-
1,10E+07	2,38E+07	-	-
1,79E+07	1,09E+07	-	-
8,83E+06	2,27E+07	1,28E+05	-
1,30E+06	7,28E+06	-	-
6,44E+06	1,49E+07	-	-
9,73E+06	1,01E+07	-	-
8,58E+06	1,35E+07	-	-
7,97E+06	2,07E+06	-	8,77E+05
5,54E+06	3,31E+06	-	-
7,06E+06	8,82E+06	-	-
6,11E+06	1,12E+07	-	-
1,53E+07	2,69E+07	-	-
3,45E+07	2,97E+07	-	-
1,69E+07	2,21E+06	-	-
8,18E+06	3,32E+06	-	-
1,44E+07	7,20E+06	-	-
1,33E+07	6,74E+06	-	-
1,21E+07	1,42E+07	-	-
1,30E+07	1,61E+07	-	-
1,74E+07	1,46E+07	-	-
6,84E+06	3,89E+06	-	-
4,61E+06	6,53E+06	-	-
2,20E+07	1,66E+07	-	-
1,44E+07	1,42E+07	-	-
7,39E+06	5,27E+06	-	-
2,72E+07	1,35E+07	-	-
1,42E+07	1,10E+07	-	-

1,01E+07	1,05E+07	-	-
1,56E+07	1,10E+07	-	-
2,46E+07	3,03E+07	-	-
1,13E+07	1,40E+07	-	-
-	2,29E+04	-	-
6,43E+06	3,44E+06	-	-
2,91E+07	1,33E+07	-	-
7,62E+06	1,79E+06	-	-
4,66E+06	8,96E+06	-	-
9,20E+06	1,08E+07	-	-
9,15E+06	5,77E+06	-	-
1,79E+07	1,02E+07	-	-
5,73E+07	6,66E+07	-	5,70E+05
9,93E+06	6,98E+06	-	-
2,77E+07	1,78E+07	-	-
2,80E+06	3,06E+06	-	-
5,87E+06	8,62E+06	-	-
2,84E+07	4,67E+07	-	-
1,12E+07	1,71E+07	-	-
8,55E+06	7,14E+06	-	-
3,94E+07	2,82E+07	-	-
9,64E+06	7,41E+06	-	-
8,46E+06	4,84E+06	1,08E+05	-
4,52E+06	9,75E+06	-	-
9,74E+06	6,64E+06	-	-
9,26E+06	1,08E+07	-	-
9,05E+06	7,43E+06	-	-
9,32E+06	1,34E+06	-	-
8,41E+06	1,16E+07	1,41E+05	-
3,22E+07	1,30E+07	-	-
1,56E+07	9,01E+06	-	-
1,14E+07	1,72E+07	-	-
1,71E+06	2,74E+04	-	-
8,49E+06	7,63E+06	-	-
1,36E+07	1,10E+07	-	-
9,33E+06	1,04E+07	-	-
1,60E+07	2,66E+07	-	-
1,07E+07	8,92E+06	-	-
5,07E+07	2,94E+07	-	-
1,87E+07	7,75E+06	-	-
1,61E+06	1,95E+06	-	-
3,52E+06	9,58E+06	-	-
7,71E+06	9,32E+06	-	-
8,98E+06	7,93E+06	-	-
2,26E+07	1,26E+07	-	-
8,12E+06	6,93E+06	-	-
3,12E+07	1,08E+07	-	-
2,42E+07	3,20E+07	-	-
8,54E+06	1,21E+07	-	-
1,69E+07	1,66E+07	-	-

1,39E+07	8,45E+06	-	-
1,13E+07	7,66E+06	-	-
2,84E+07	2,35E+07	-	-
1,91E+07	1,62E+07	-	-
3,39E+07	3,01E+07	-	-
8,25E+06	7,04E+06	-	-
6,85E+06	3,02E+07	-	-
2,83E+07	5,31E+06	-	-
5,72E+07	3,01E+07	-	-
2,17E+07	2,57E+07	-	-
8,68E+06	1,01E+07	-	-
1,16E+07	5,78E+06	-	-
2,30E+07	3,90E+07	-	-
4,60E+06	8,87E+06	-	-
1,21E+07	2,95E+07	-	-
6,35E+06	1,75E+07	-	-
2,85E+06	3,02E+06	-	-
1,31E+07	8,23E+06	-	-
7,13E+06	1,17E+06	-	-
8,97E+07	5,69E+07	-	1,00E+06
1,05E+07	7,56E+06	-	-
8,32E+06	8,33E+06	-	-
5,38E+06	2,51E+07	-	-
1,39E+07	1,81E+07	-	-
6,37E+07	5,17E+07	-	-
1,22E+07	1,01E+07	-	-
8,03E+07	6,95E+07	-	2,05E+06
3,45E+06	1,04E+07	-	-
1,84E+07	1,70E+07	-	-
1,07E+07	1,54E+07	-	-
9,59E+06	1,48E+07	1,73E+05	-
1,13E+07	6,56E+06	-	-
3,46E+07	4,39E+07	-	-
5,69E+06	8,74E+06	-	-
1,37E+07	2,13E+07	-	-
9,12E+06	7,10E+06	-	-
1,22E+07	4,95E+06	-	-
6,85E+06	1,11E+07	-	-
1,65E+07	2,32E+07	-	-
2,28E+07	1,97E+07	-	-
7,89E+06	8,26E+06	-	-
1,25E+07	2,61E+07	-	-
7,49E+06	6,45E+06	-	-
3,33E+07	1,97E+07	-	-
1,80E+07	2,16E+07	-	-
2,94E+07	2,48E+07	-	5,63E+05
3,71E+06	7,62E+06	-	-
4,11E+06	7,80E+06	-	-
1,70E+07	1,57E+07	-	-
1,86E+07	6,35E+06	-	-

2,30E+07	1,55E+07	-	-
2,54E+07	1,06E+07	-	-
9,18E+06	8,43E+06	-	-
5,58E+06	4,39E+06	-	5,88E+05
1,20E+07	1,13E+07	-	-
1,12E+07	9,12E+06	-	-
1,43E+07	1,38E+07	-	-
1,23E+07	1,04E+07	-	-
1,60E+07	1,12E+07	-	-
1,24E+07	1,30E+07	-	-
2,61E+07	5,57E+06	-	-
3,10E+07	3,55E+07	-	-
1,19E+07	7,31E+06	-	-
2,40E+07	2,04E+07	-	-
7,84E+06	1,59E+07	-	-
5,45E+06	8,63E+06	-	-
9,98E+06	8,61E+06	-	-
1,56E+07	1,57E+07	-	-
4,47E+07	5,26E+07	-	-
9,50E+06	4,79E+06	-	-

Stock Solution Okara	Stock Solution Okara US					
-	-	C	7	H	12	N
8,05E+06	6,45E+06	C	7	H	14	N
5,75E+06	6,78E+06	C	6	H	12	N
-	-	C	8	H	14	N
7,78E+06	5,04E+06	C	8	H	16	N
1,82E+07	1,94E+07	C	8	H	16	N
-	-	C	8	H	16	N
5,81E+06	4,98E+06	C	8	H	16	N
3,98E+07	2,18E+07	C	8	H	16	N
1,19E+07	9,08E+06	C	7	H	14	N
-	-	C	8	H	14	N
7,63E+06	6,21E+06	C	9	H	18	N
8,20E+07	6,30E+07	C	9	H	18	N
2,92E+07	2,68E+07	C	9	H	18	N
1,87E+07	1,38E+07	C	9	H	18	N
1,55E+07	1,62E+07	C	8	H	16	N
-	-	C	10	H	16	N
-	-	C	10	H	18	N
-	-	C	9	H	16	N
2,99E+07	1,88E+07	C	10	H	20	N
9,24E+06	8,35E+06	C	8	H	14	N
8,11E+07	5,07E+07	C	9	H	18	N
1,28E+07	1,13E+07	C	9	H	18	N
1,50E+07	9,98E+06	C	9	H	18	N
1,27E+07	9,06E+06	C	9	H	18	N
2,24E+07	1,70E+07	C	11	H	14	N
4,24E+06	5,57E+06	C	11	H	14	N
1,23E+07	1,20E+07	C	9	H	14	N
-	-	C	11	H	20	N
-	-	C	11	H	20	N
-	-	C	9	H	15	N
-	-	C	11	H	22	N
1,08E+08	9,31E+07	C	11	H	22	N
1,08E+07	1,33E+07	C	11	H	22	N
7,04E+06	1,00E+07	C	11	H	22	N
1,14E+07	1,18E+07	C	9	H	17	N
1,79E+07	1,60E+07	C	8	H	17	N
5,02E+06	5,31E+06	C	9	H	16	N
1,02E+07	8,75E+06	C	10	H	20	N
1,16E+08	8,01E+07	C	10	H	20	N
2,06E+07	2,15E+07	C	9	H	19	N
1,03E+07	1,06E+07	C	8	H	14	N
5,29E+07	4,07E+07	C	12	H	16	N
-	-	C	12	H	16	N
1,01E+07	1,34E+07	C	11	H	14	N
2,59E+06	5,08E+06	C	11	H	14	N
-	-	C	11	H	21	N
1,24E+08	9,71E+07	C	12	H	24	N
7,00E+07	5,46E+07	C	12	H	24	N

1,05E+07	6,57E+06	**C**	12	**H**	24	**N**
4,67E+06	4,94E+06	**C**	10	**H**	19	**N**
1,25E+07	1,49E+07	**C**	10	**H**	19	**N**
3,34E+07	1,72E+07	**C**	10	**H**	19	**N**
7,58E+06	3,40E+06	**C**	10	**H**	19	**N**
7,89E+06	1,04E+07	**C**	10	**H**	19	**N**
1,36E+07	1,19E+07	**C**	9	**H**	19	**N**
-	-	**C**	9	**H**	19	**N**
5,71E+06	7,89E+06	**C**	11	**H**	23	**N**
1,80E+07	2,37E+07	**C**	10	**H**	18	**N**
3,17E+07	1,90E+07	**C**	10	**H**	18	**N**
2,43E+06	2,04E+06	**C**	10	**H**	21	**N**
5,67E+06	4,83E+06	**C**	9	**H**	16	**N**
1,57E+07	1,10E+07	**C**	9	**H**	18	**N**
1,32E+07	8,41E+06	**C**	12	**H**	16	**N**
9,33E+05	1,05E+06	**C**	12	**H**	16	**N**
-	-	**C**	11	**H**	16	**N**
3,57E+06	4,93E+06	**C**	11	**H**	18	**N**
3,65E+07	4,45E+07	**C**	11	**H**	21	**N**
4,39E+07	2,79E+07	**C**	11	**H**	21	**N**
1,23E+07	7,04E+06	**C**	11	**H**	21	**N**
3,78E+07	4,91E+07	**C**	12	**H**	25	**N**
1,22E+07	1,04E+07	**C**	12	**H**	25	**N**
1,72E+07	1,32E+07	**C**	11	**H**	20	**N**
2,34E+07	1,85E+07	**C**	11	**H**	20	**N**
1,16E+07	1,16E+07	**C**	11	**H**	20	**N**
9,32E+06	5,67E+06	**C**	13	**H**	15	**N**
3,78E+07	2,80E+07	**C**	9	**H**	19	**N**
2,44E+06	1,92E+06	**C**	14	**H**	18	**N**
6,03E+06	7,55E+06	**C**	11	**H**	22	**N**
1,35E+07	8,67E+06	**C**	11	**H**	22	**N**
7,49E+06	5,44E+06	**C**	11	**H**	22	**N**
8,41E+07	6,39E+07	**C**	14	**H**	20	**N**
5,05E+07	3,74E+07	**C**	13	**H**	18	**N**
3,94E+07	3,29E+07	**C**	12	**H**	16	**N**
3,02E+07	1,77E+07	**C**	12	**H**	20	**N**
7,99E+06	4,50E+06	**C**	12	**H**	20	**N**
1,02E+06	1,88E+06	**C**	11	**H**	21	**N**
1,49E+07	1,21E+07	**C**	11	**H**	23	**N**
7,11E+06	4,59E+06	**C**	11	**H**	23	**N**
-	-	**C**	11	**H**	22	**N**
9,72E+06	1,01E+07	**C**	14	**H**	17	**N**
4,21E+06	5,93E+06	**C**	11	**H**	21	**N**
6,09E+06	7,90E+06	**C**	10	**H**	21	**N**
1,40E+07	1,43E+07	**C**	10	**H**	16	**N**
5,22E+06	7,39E+06	**C**	10	**H**	19	**N**
-	-	**C**	14	**H**	18	**N**
8,81E+07	6,40E+07	**C**	15	**H**	22	**N**
4,75E+07	3,10E+07	**C**	15	**H**	22	**N**
1,61E+08	1,30E+08	**C**	15	**H**	22	**N**

1,32E+07	1,25E+07	**C**	13	**H**	17	**N**
4,95E+07	3,34E+07	**C**	13	**H**	17	**N**
7,55E+06	1,09E+07	**C**	13	**H**	16	**N**
1,17E+07	8,05E+06	**C**	14	**H**	20	**N**
2,20E+06	1,76E+06	**C**	14	**H**	20	**N**
2,93E+07	2,16E+07	**C**	13	**H**	18	**N**
9,71E+06	1,12E+07	**C**	11	**H**	16	**N**
2,29E+07	1,88E+07	**C**	13	**H**	23	**N**
1,29E+07	7,33E+06	**C**	13	**H**	25	**N**
4,05E+07	4,57E+07	**C**	12	**H**	25	**N**
8,65E+07	9,69E+07	**C**	12	**H**	25	**N**
1,63E+08	1,19E+08	**C**	12	**H**	25	**N**
-	-	**C**	10	**H**	20	**N**
-	-	**C**	10	**H**	20	**N**
2,03E+07	1,50E+07	**C**	14	**H**	17	**N**
8,67E+06	6,99E+06	**C**	14	**H**	19	**N**
2,80E+07	2,31E+07	**C**	15	**H**	23	**N**
1,87E+07	1,93E+07	**C**	15	**H**	23	**N**
1,78E+07	1,61E+07	**C**	14	**H**	18	**N**
4,21E+06	4,19E+06	**C**	14	**H**	18	**N**
5,79E+07	5,24E+07	**C**	15	**H**	22	**N**
3,56E+07	3,90E+07	**C**	15	**H**	22	**N**
3,19E+07	2,32E+07	**C**	15	**H**	22	**N**
6,91E+06	6,12E+06	**C**	13	**H**	16	**N**
6,96E+06	5,66E+06	**C**	14	**H**	20	**N**
-	-	**C**	14	**H**	25	**N**
4,64E+06	4,91E+06	**C**	14	**H**	27	**N**
7,39E+06	4,42E+06	**C**	14	**H**	27	**N**
5,06E+07	4,83E+07	**C**	15	**H**	18	**N**
3,54E+06	4,29E+06	**C**	15	**H**	18	**N**
1,23E+07	7,27E+06	**C**	12	**H**	22	**N**
4,97E+06	3,62E+06	**C**	11	**H**	22	**N**
1,72E+07	1,76E+07	**C**	11	**H**	22	**N**
8,64E+06	1,74E+06	**C**	13	**H**	26	**N**
6,28E+06	7,49E+06	**C**	12	**H**	26	**N**
2,56E+06	7,49E+06	**C**	12	**H**	21	**N**
9,07E+06	1,22E+07	**C**	11	**H**	21	**N**
5,32E+06	2,70E+06	**C**	16	**H**	21	**N**
2,21E+07	3,46E+07	**C**	15	**H**	19	**N**
5,38E+06	5,56E+06	**C**	11	**H**	23	**N**
1,34E+07	1,09E+07	**C**	14	**H**	19	**N**
2,60E+07	1,93E+07	**C**	15	**H**	23	**N**
7,28E+06	8,51E+06	**C**	14	**H**	18	**N**
1,85E+06	2,09E+06	**C**	12	**H**	21	**N**
7,69E+06	8,28E+06	**C**	14	**H**	20	**N**
5,89E+07	4,85E+07	**C**	18	**H**	20	**N**
3,02E+06	5,56E+06	**C**	13	**H**	21	**N**
1,99E+06	2,58E+06	**C**	14	**H**	25	**N**
2,64E+07	1,61E+07	**C**	15	**H**	29	**N**
9,16E+06	5,88E+06	**C**	12	**H**	24	**N**

4,06E+06	5,74E+06	**C**	14	**H**	28	**N**
7,04E+06	3,69E+06	**C**	13	**H**	23	**N**
1,35E+07	8,07E+06	**C**	17	**H**	23	**N**
1,40E+07	1,35E+07	**C**	17	**H**	23	**N**
8,76E+06	6,10E+06	**C**	17	**H**	23	**N**
5,53E+06	6,19E+06	**C**	14	**H**	27	**N**
9,36E+06	1,19E+07	**C**	14	**H**	27	**N**
6,63E+06	5,41E+06	**C**	15	**H**	18	**N**
3,33E+06	3,96E+06	**C**	12	**H**	21	**N**
6,57E+06	2,05E+06	**C**	13	**H**	25	**N**
1,29E+07	9,75E+06	**C**	13	**H**	25	**N**
6,43E+07	6,34E+07	**C**	15	**H**	23	**N**
1,46E+07	1,75E+07	**C**	15	**H**	23	**N**
1,28E+07	1,19E+07	**C**	18	**H**	20	**N**
1,68E+07	2,59E+07	**C**	18	**H**	20	**N**
2,14E+07	1,05E+07	**C**	15	**H**	27	**N**
3,39E+07	1,53E+07	**C**	16	**H**	31	**N**
3,86E+06	5,60E+06	**C**	14	**H**	26	**N**
1,22E+07	6,05E+06	**C**	14	**H**	26	**N**
6,47E+06	1,22E+07	**C**	14	**H**	26	**N**
1,24E+07	1,11E+07	**C**	15	**H**	30	**N**
8,96E+06	5,75E+06	**C**	14	**H**	25	**N**
9,92E+06	1,09E+07	**C**	15	**H**	29	**N**
9,55E+06	1,60E+07	**C**	15	**H**	29	**N**
9,92E+06	1,09E+07	**C**	15	**H**	29	**N**
7,59E+06	1,65E+07	**C**	15	**H**	29	**N**
9,91E+05	4,26E+06	**C**	16	**H**	20	**N**
5,26E+06	1,01E+07	**C**	17	**H**	24	**N**
5,13E+06	5,62E+06	**C**	14	**H**	28	**N**
6,45E+06	8,06E+06	**C**	16	**H**	19	**N**
6,74E+06	1,42E+06	**C**	17	**H**	23	**N**
-	-	**C**	12	**H**	21	**N**
6,01E+06	6,02E+06	**C**	17	**H**	25	**N**
5,06E+06	7,64E+06	**C**	17	**H**	25	**N**
1,06E+07	1,53E+07	**C**	15	**H**	23	**N**
2,44E+07	1,69E+07	**C**	15	**H**	23	**N**
1,29E+07	1,35E+06	**C**	15	**H**	25	**N**
5,70E+06	2,16E+06	**C**	17	**H**	19	**N**
1,21E+07	5,02E+06	**C**	15	**H**	26	**N**
1,14E+07	4,73E+06	**C**	17	**H**	33	**N**
1,04E+07	9,52E+06	**C**	17	**H**	33	**N**
1,21E+07	1,25E+07	**C**	18	**H**	20	**N**
1,70E+07	1,18E+07	**C**	15	**H**	28	**N**
5,45E+06	2,56E+06	**C**	15	**H**	28	**N**
4,25E+06	4,24E+06	**C**	14	**H**	28	**N**
1,44E+07	8,72E+06	**C**	14	**H**	28	**N**
9,14E+06	7,12E+06	**C**	14	**H**	28	**N**
5,90E+06	3,76E+06	**C**	15	**H**	27	**N**
2,29E+07	1,14E+07	**C**	16	**H**	31	**N**
1,19E+07	7,57E+06	**C**	16	**H**	31	**N**

5,65E+06	5,75E+06	**C**	15	**H**	30	**N**
2,62E+06	9,21E+06	**C**	15	**H**	30	**N**
1,48E+07	2,10E+07	**C**	14	**H**	25	**N**
9,47E+06	9,65E+06	**C**	14	**H**	25	**N**
2,20E+06	1,51E+06	**C**	14	**H**	25	**N**
5,97E+06	2,51E+06	**C**	16	**H**	21	**N**
2,45E+07	9,27E+06	**C**	17	**H**	25	**N**
7,10E+06	1,25E+06	**C**	15	**H**	25	**N**
3,82E+06	6,02E+06	**C**	16	**H**	27	**N**
8,13E+06	7,56E+06	**C**	18	**H**	35	**N**
7,42E+06	3,76E+06	**C**	18	**H**	35	**N**
6,64E+06	9,21E+06	**C**	14	**H**	26	**N**
4,89E+07	4,50E+07	**C**	16	**H**	30	**N**
8,56E+06	4,69E+06	**C**	16	**H**	30	**N**
2,67E+07	1,54E+07	**C**	16	**H**	30	**N**
2,02E+06	9,78E+05	**C**	16	**H**	30	**N**
5,13E+06	6,42E+06	**C**	16	**H**	30	**N**
3,21E+06	2,66E+06	**C**	15	**H**	30	**N**
7,35E+06	1,09E+07	**C**	15	**H**	30	**N**
7,75E+06	4,84E+06	**C**	15	**H**	30	**N**
3,15E+07	1,79E+07	**C**	17	**H**	34	**N**
8,39E+06	5,68E+06	**C**	14	**H**	25	**N**
7,13E+06	3,17E+06	**C**	16	**H**	29	**N**
3,95E+06	6,94E+06	**C**	16	**H**	29	**N**
9,44E+06	4,44E+06	**C**	16	**H**	29	**N**
9,34E+06	8,97E+06	**C**	15	**H**	28	**N**
1,06E+07	4,70E+06	**C**	14	**H**	28	**N**
1,00E+07	1,20E+06	**C**	19	**H**	27	**N**
1,13E+07	9,85E+06	**C**	13	**H**	21	**N**
2,94E+07	1,02E+07	**C**	19	**H**	29	**N**
1,51E+07	6,74E+06	**C**	18	**H**	27	**N**
8,29E+06	9,87E+06	**C**	17	**H**	27	**N**
2,64E+06	3,35E+06	**C**	20	**H**	21	**N**
6,07E+06	5,31E+06	**C**	16	**H**	32	**N**
1,08E+07	6,56E+06	**C**	18	**H**	36	**N**
7,65E+06	6,60E+06	**C**	18	**H**	23	**N**
1,35E+07	2,00E+07	**C**	15	**H**	27	**N**
9,02E+06	6,21E+06	**C**	17	**H**	31	**N**
4,04E+07	1,84E+07	**C**	16	**H**	31	**N**
3,39E+06	5,09E+06	**C**	16	**H**	31	**N**
5,06E+06	3,75E+06	**C**	15	**H**	26	**N**
-	-	**C**	14	**H**	26	**N**
7,14E+06	6,65E+06	**C**	19	**H**	26	**N**
5,86E+06	4,38E+06	**C**	16	**H**	30	**N**
1,71E+07	9,12E+06	**C**	15	**H**	30	**N**
3,95E+05	3,09E+04	**C**	15	**H**	30	**N**
4,62E+05	5,92E+05	**C**	15	**H**	30	**N**
1,80E+07	1,91E+07	**C**	15	**H**	30	**N**
7,04E+06	7,42E+06	**C**	15	**H**	25	**N**
1,17E+07	9,93E+06	**C**	19	**H**	27	**N**

1,21E+07	5,80E+06	**C**	20	**H**	31	**N**
9,70E+06	5,16E+06	**C**	20	**H**	31	**N**
2,21E+07	1,65E+07	**C**	20	**H**	31	**N**
1,64E+07	1,13E+07	**C**	18	**H**	25	**N**
2,67E+07	2,05E+07	**C**	18	**H**	25	**N**
7,11E+06	4,79E+06	**C**	19	**H**	29	**N**
5,02E+06	1,53E+07	**C**	16	**H**	23	**N**
2,21E+07	4,08E+06	**C**	16	**H**	25	**N**
5,11E+07	2,35E+07	**C**	17	**H**	34	**N**
7,60E+06	7,08E+06	**C**	17	**H**	34	**N**
2,06E+06	8,80E+05	**C**	17	**H**	34	**N**
9,13E+06	3,75E+06	**C**	17	**H**	34	**N**
1,65E+07	2,43E+07	**C**	17	**H**	33	**N**
3,31E+06	4,93E+06	**C**	17	**H**	33	**N**
1,04E+07	2,13E+07	**C**	16	**H**	27	**N**
4,80E+06	1,16E+07	**C**	17	**H**	34	**N**
2,26E+06	1,95E+06	**C**	20	**H**	29	**N**
1,17E+07	5,69E+06	**C**	21	**H**	33	**N**
6,53E+06	8,35E+05	**C**	19	**H**	27	**N**
7,61E+07	4,00E+07	**C**	19	**H**	27	**N**
8,83E+06	5,44E+06	**C**	20	**H**	31	**N**
7,04E+06	5,87E+06	**C**	21	**H**	25	**N**
3,42E+06	1,69E+07	**C**	17	**H**	29	**N**
7,18E+06	1,12E+07	**C**	16	**H**	29	**N**
4,66E+07	3,21E+07	**C**	18	**H**	36	**N**
1,00E+07	6,09E+06	**C**	18	**H**	36	**N**
6,51E+07	5,71E+07	**C**	16	**H**	31	**N**
2,56E+06	6,78E+06	**C**	16	**H**	30	**N**
1,53E+07	1,12E+07	**C**	16	**H**	30	**N**
2,45E+05	5,30E+05	**C**	16	**H**	28	**N**
1,00E+07	1,26E+07	**C**	15	**H**	23	**N**
8,75E+06	4,14E+06	**C**	21	**H**	34	**N**
2,10E+06	1,11E+06	**C**	21	**H**	34	**N**
4,83E+06	5,37E+06	**C**	20	**H**	29	**N**
1,28E+07	1,53E+07	**C**	20	**H**	29	**N**
7,82E+06	4,06E+06	**C**	21	**H**	33	**N**
1,03E+07	3,44E+06	**C**	21	**H**	33	**N**
5,70E+06	8,50E+06	**C**	18	**H**	24	**N**
1,37E+07	1,44E+07	**C**	21	**H**	29	**N**
1,87E+07	1,35E+07	**C**	17	**H**	33	**N**
7,15E+06	6,02E+06	**C**	19	**H**	24	**N**
9,74E+06	1,84E+07	**C**	17	**H**	32	**N**
5,04E+06	3,94E+06	**C**	17	**H**	32	**N**
2,45E+07	1,11E+07	**C**	20	**H**	32	**N**
1,36E+07	1,48E+07	**C**	21	**H**	34	**N**
2,52E+07	1,78E+07	**C**	19	**H**	25	**N**
3,30E+06	5,54E+06	**C**	20	**H**	29	**N**
2,84E+06	4,78E+06	**C**	18	**H**	28	**N**
1,39E+07	1,06E+07	**C**	23	**H**	27	**N**
1,59E+07	4,70E+06	**C**	24	**H**	31	**N**

1,89E+07	1,08E+07	**C**	22	**H**	26	**N**
2,20E+07	7,34E+06	**C**	23	**H**	34	**N**
7,66E+06	5,61E+06	**C**	23	**H**	34	**N**
1,25E+06	1,72E+06	**C**	16	**H**	28	**N**
9,57E+06	6,97E+06	**C**	20	**H**	31	**N**
9,16E+06	5,99E+06	**C**	24	**H**	32	**N**
1,24E+07	1,01E+07	**C**	23	**H**	27	**N**
1,24E+07	8,55E+06	**C**	18	**H**	37	**N**
1,46E+07	8,11E+06	**C**	18	**H**	37	**N**
8,94E+06	7,93E+06	**C**	20	**H**	31	**N**
2,09E+07	3,92E+06	**C**	20	**H**	31	**N**
2,93E+07	2,32E+07	**C**	20	**H**	30	**N**
9,02E+06	4,67E+06	**C**	21	**H**	34	**N**
1,92E+07	1,32E+07	**C**	19	**H**	28	**N**
6,65E+06	1,26E+07	**C**	21	**H**	27	**N**
2,82E+06	2,73E+06	**C**	21	**H**	35	**N**
7,23E+06	5,62E+06	**C**	20	**H**	30	**N**
1,31E+07	1,07E+07	**C**	24	**H**	28	**N**
3,44E+07	3,43E+07	**C**	24	**H**	32	**N**
8,84E+06	3,27E+06	**C**	25	**H**	30	**N**

					Calculated Molecular Weight
2	**O**	3			172,084793
2	**O**	3			174,100443
2	**O**	4			176,079708
2	**O**	3			186,100443
2	**O**	3			188,116093
2	**O**	3			188,116093
2	**O**	3			188,116093
2	**O**	3			188,116093
2	**O**	3			188,116093
2	**O**	4			190,095358
2	**O**	4			202,095358
2	**O**	3			202,131743
2	**O**	3			202,131743
2	**O**	3			202,131743
2	**O**	3			202,131743
2	**O**	4			204,111008
2	**O**	3			212,116093
2	**O**	3			214,131743
2	**O**	4			216,111008
2	**O**	3			216,147393
2	**O**	5			218,090273
2	**O**	4			218,126658
2	**O**	4			218,126658
2	**O**	4			218,126658
2	**O**	4			218,126658
2	**O**	3			222,100443
2	**O**	3			222,100443
4	**O**	3			226,106591
2	**O**	3			228,147393
2	**O**	3			228,147393
3	**O**	4			229,106257
2	**O**	3			230,163043
2	**O**	3			230,163043
2	**O**	3			230,163043
2	**O**	3			230,163043
3	**O**	4			231,121907
5	**O**	3			231,13314
2	**O**	5			232,105923
2	**O**	4			232,142308
2	**O**	4			232,142308
3	**O**	4			233,137557
2	**O**	6			234,085188
2	**O**	3			236,116093
2	**O**	3			236,116093
2	**O**	4			238,095358
2	**O**	4			238,095358
3	**O**	3			243,158292
2	**O**	3			244,178693
2	**O**	3			244,178693

2	**O**	3			244,178693
3	**O**	4			245,137557
3	**O**	4			245,137557
3	**O**	4			245,137557
3	**O**	4			245,137557
3	**O**	4			245,137557
5	**O**	3			245,14879
5	**O**	3			245,14879
3	**O**	3			245,173942
2	**O**	5			246,121573
2	**O**	5			246,121573
3	**O**	4			247,153207
2	**O**	6			248,100838
2	**O**	4	**S**	1	250,09873
2	**O**	4			252,111008
2	**O**	4			252,111008
4	**O**	3			252,122241
4	**O**	3			254,137891
3	**O**	4			259,153207
3	**O**	4			259,153207
3	**O**	4			259,153207
3	**O**	3			259,189592
3	**O**	3			259,189592
2	**O**	5			260,137223
2	**O**	5			260,137223
2	**O**	5			260,137223
3	**O**	3			261,111342
5	**O**	4			261,143705
2	**O**	3			262,131743
2	**O**	3	**S**	1	262,135115
2	**O**	3	**S**	1	262,135115
2	**O**	3	**S**	1	262,135115
2	**O**	3			264,147393
2	**O**	4			266,126658
2	**O**	5			268,105923
4	**O**	3			268,153541
4	**O**	3			268,153541
5	**O**	3			271,16444
5	**O**	3			273,18009
5	**O**	3			273,18009
4	**O**	4			274,164106
3	**O**	3			275,126992
3	**O**	5			275,148122
5	**O**	4			275,159355
2	**O**	7			276,095753
3	**O**	4	**S**	1	277,109629
2	**O**	4			278,126658
2	**O**	3			278,163043
2	**O**	3			278,163043
2	**O**	3			278,163043

3	**O**	4			279,121907
3	**O**	4			279,121907
2	**O**	5			280,105923
2	**O**	4			280,142308
2	**O**	4			280,142308
2	**O**	5			282,121573
4	**O**	5			284,112071
3	**O**	4			285,168857
3	**O**	4			287,184507
5	**O**	3			287,19574
5	**O**	3			287,19574
5	**O**	3			287,19574
6	**O**	4			288,154604
6	**O**	4			288,154604
3	**O**	4			291,121907
3	**O**	4			293,137557
3	**O**	3			293,173942
3	**O**	3			293,173942
2	**O**	5			294,121573
2	**O**	5			294,121573
2	**O**	4			294,157958
2	**O**	4			294,157958
2	**O**	4			294,157958
2	**O**	6			296,100838
2	**O**	3	**S**	1	296,119465
3	**O**	4			299,184507
3	**O**	4			301,200157
3	**O**	4			301,200157
4	**O**	3			302,137891
4	**O**	3			302,137891
4	**O**	5			302,159021
6	**O**	4			302,170254
6	**O**	4			302,170254
4	**O**	4			302,195406
6	**O**	3			302,206639
3	**O**	4	**S**	1	303,125279
5	**O**	5			303,15427
3	**O**	3			303,158292
3	**O**	4			305,137557
5	**O**	3	**S**	1	305,152162
3	**O**	5			309,132472
3	**O**	4			309,168857
2	**O**	6			310,116488
7	**O**	3			311,170588
2	**O**	4	**S**	1	312,11438
2	**O**	3			312,147393
3	**O**	6			315,143037
3	**O**	5			315,179422
3	**O**	4			315,215807
6	**O**	4			316,185904

4	**O**	4			316,211056
3	**O**	6			317,158687
3	**O**	3			317,173942
3	**O**	3			317,173942
3	**O**	3			317,173942
3	**O**	5			317,195072
3	**O**	5			317,195072
4	**O**	4			318,132806
3	**O**	7			319,137952
3	**O**	6			319,174337
3	**O**	6			319,174337
5	**O**	3			321,18009
5	**O**	3			321,18009
2	**O**	4			328,142308
2	**O**	4			328,142308
3	**O**	5			329,195072
3	**O**	4			329,231457
4	**O**	5			330,190321
4	**O**	5			330,190321
4	**O**	5			330,190321
4	**O**	4			330,226706
3	**O**	6			331,174337
3	**O**	5			331,210722
3	**O**	5			331,210722
3	**O**	5			331,210722
3	**O**	5			331,210722
4	**O**	4			332,148456
4	**O**	3			332,184841
4	**O**	5			332,205971
3	**O**	5			333,132472
3	**O**	4			333,168857
3	**O**	8			335,132867
3	**O**	4			335,184507
3	**O**	4			335,184507
5	**O**	4			337,175005
5	**O**	4			337,175005
5	**O**	4			339,190655
5	**O**	3			341,14879
4	**O**	5			342,190321
3	**O**	4			343,247107
3	**O**	4			343,247107
2	**O**	5			344,137223
4	**O**	5			344,205971
4	**O**	5			344,205971
6	**O**	4			344,217204
6	**O**	4			344,217204
6	**O**	4			344,217204
3	**O**	6			345,189987
3	**O**	5			345,226372
3	**O**	5			345,226372

4	**O**	5			346,221621
4	**O**	5			346,221621
3	**O**	7			347,169252
3	**O**	7			347,169252
3	**O**	7			347,169252
3	**O**	6			351,143037
3	**O**	5			351,179422
5	**O**	5			355,18557
3	**O**	6			357,189987
3	**O**	4			357,262757
3	**O**	4			357,262757
6	**O**	5			358,196469
4	**O**	5			358,221621
4	**O**	5			358,221621
4	**O**	5			358,221621
4	**O**	5			358,221621
4	**O**	5			358,221621
6	**O**	4			358,232854
6	**O**	4			358,232854
6	**O**	4			358,232854
4	**O**	4			358,258006
5	**O**	6			359,180485
3	**O**	6			359,205637
3	**O**	6			359,205637
3	**O**	6			359,205637
4	**O**	6			360,200886
6	**O**	5			360,212119
3	**O**	4			361,200157
3	**O**	9			363,127782
3	**O**	4			363,215807
3	**O**	5			365,195072
5	**O**	4			365,206305
3	**O**	4			367,153207
6	**O**	4			372,248504
4	**O**	4			372,273656
5	**O**	4			373,175005
5	**O**	6			373,196135
3	**O**	6			373,221287
5	**O**	5			373,23252
5	**O**	5			373,23252
4	**O**	7			374,180151
6	**O**	6			374,191384
4	**O**	4			374,195406
4	**O**	6			374,216536
6	**O**	5			374,227769
6	**O**	5			374,227769
6	**O**	5			374,227769
6	**O**	5			374,227769
3	**O**	8			375,164167
3	**O**	5			377,195072

3	**O**	4			377,231457
3	**O**	4			377,231457
3	**O**	4			377,231457
3	**O**	6			379,174337
3	**O**	6			379,174337
3	**O**	5			379,210722
5	**O**	6			381,164835
5	**O**	6			383,180485
6	**O**	4			386,264154
6	**O**	4			386,264154
6	**O**	4			386,264154
6	**O**	4			386,264154
5	**O**	5			387,24817
5	**O**	5			387,24817
3	**O**	8			389,179817
4	**O**	4	**S**	1	390,230078
3	**O**	5			391,210722
3	**O**	4			391,247107
3	**O**	6			393,189987
3	**O**	6			393,189987
3	**O**	5			393,226372
3	**O**	5			399,179422
5	**O**	4	**S**	1	399,194027
7	**O**	5			399,223018
6	**O**	4			400,279804
6	**O**	4			400,279804
7	**O**	5			401,238668
6	**O**	6			402,222684
6	**O**	6			402,222684
4	**O**	8			404,190716
3	**O**	10			405,138347
4	**O**	4			406,258006
4	**O**	4			406,258006
3	**O**	6			407,205637
3	**O**	6			407,205637
3	**O**	5			407,242022
3	**O**	5			407,242022
4	**O**	7			408,164501
5	**O**	4			415,221955
7	**O**	5			415,254318
6	**O**	5			416,180819
6	**O**	6			416,238334
6	**O**	6			416,238334
6	**O**	4			420,248504
4	**O**	5			422,252921
3	**O**	8			423,164167
3	**O**	7			423,200552
6	**O**	6			424,207034
3	**O**	5			425,195072
3	**O**	4			425,231457

4	**O**	5			426,190321
4	**O**	4			430,258006
4	**O**	4			430,258006
6	**O**	8			432,196864
5	**O**	6			437,227435
4	**O**	4			440,242356
3	**O**	6			441,189987
9	**O**	4			443,296851
9	**O**	4			443,296851
7	**O**	5			449,238668
7	**O**	5			449,238668
6	**O**	6			450,222684
6	**O**	5			450,259069
6	**O**	7			452,201949
5	**O**	7			461,19105
7	**O**	5			465,269968
6	**O**	7			466,217599
4	**O**	6			468,200886
6	**O**	5			484,243419
4	**O**	5	**S**	1	498,193693

Calculated m/z	Experimental Molecular Weight	Experimental m/z	Δmass (ppm)
173,092069	172,08426	173,091536	-3,08
175,107719	174,09984	175,107116	-3,44
177,086984	176,07908	177,086356	-3,55
187,107719	186,09982	187,107096	-3,33
189,123369	188,11534	189,122616	-3,98
189,123369	188,11539	189,122666	-3,72
189,123369	188,11544	189,122716	-3,45
189,123369	188,11544	189,122716	-3,45
189,123369	188,11546	189,122736	-3,35
191,102634	190,0946	191,101876	-3,97
203,102634	202,09474	203,102016	-3,04
203,139019	202,13106	203,138336	-3,36
203,139019	202,13106	203,138336	-3,36
203,139019	202,13107	203,138346	-3,31
203,139019	202,13109	203,138366	-3,21
205,118284	204,11034	205,117616	-3,26
213,123369	212,11531	213,122586	-3,67
215,139019	214,131	215,138276	-3,45
217,118284	216,11035	217,117626	-3,03
217,154669	216,14666	217,153936	-3,38
219,097549	218,0894	219,096676	-3,98
219,133934	218,12589	219,133166	-3,50
219,133934	218,1259	219,133176	-3,46
219,133934	218,12593	219,133206	-3,32
219,133934	218,12593	219,133206	-3,32
223,107719	222,0997	223,106976	-3,33
223,107719	222,0997	223,106976	-3,33
227,113867	226,10578	227,113056	-3,57
229,154669	228,14656	229,153836	-3,64
229,154669	228,14657	229,153846	-3,59
230,113533	229,10552	230,112796	-3,20
231,170319	230,16195	231,169226	-4,73
231,170319	230,16195	231,169226	-4,73
231,170319	230,16196	231,169236	-4,68
231,170319	230,16197	231,169246	-4,64
232,129183	231,12106	232,128336	-3,65
232,140416	231,13228	232,139556	-3,70
233,113199	232,10513	233,112406	-3,40
233,149584	232,14133	233,148606	-4,19
233,149584	232,14138	233,148656	-3,98
234,144833	233,13666	234,143936	-3,83
235,092464	234,08426	235,091536	-3,95
237,123369	236,11521	237,122486	-3,72
237,123369	236,11522	237,122496	-3,68
239,102634	238,0944	239,101676	-4,01
239,102634	238,09443	239,101706	-3,88
244,165568	243,15721	244,164486	-4,43
245,185969	244,17764	245,184916	-4,29
245,185969	244,17764	245,184916	-4,29

245,185969	244,17774	245,185016	-3,89
246,144833	245,13656	246,143836	-4,05
246,144833	245,13657	246,143846	-4,01
246,144833	245,1366	246,143876	-3,89
246,144833	245,13662	246,143896	-3,81
246,144833	245,13664	246,143916	-3,73
246,156066	245,14771	246,154986	-4,39
246,156066	245,14773	246,155006	-4,31
246,181218	245,17288	246,180156	-4,31
247,128849	246,12056	247,127836	-4,10
247,128849	246,12064	247,127916	-3,78
248,160483	247,15224	248,159516	-3,90
249,108114	248,09979	249,107066	-4,21
251,106006	250,09777	251,105046	-3,82
253,118284	252,10994	253,117216	-4,22
253,118284	252,11	253,117276	-3,98
253,129517	252,12112	253,128396	-4,43
255,145167	254,13681	255,144086	-4,24
260,160483	259,15212	260,159396	-4,18
260,160483	259,15214	260,159416	-4,10
260,160483	259,1522	260,159476	-3,87
260,196868	259,18857	260,195846	-3,93
260,196868	259,18858	260,195856	-3,89
261,144499	260,13619	261,143466	-3,96
261,144499	260,1362	261,143476	-3,92
261,144499	260,13625	261,143526	-3,73
262,118618	261,11036	262,117636	-3,75
262,150981	261,14246	262,149736	-4,75
263,139019	262,13066	263,137936	-4,12
263,142391	262,13411	263,141386	-3,82
263,142391	262,13411	263,141386	-3,82
263,142391	262,13415	263,141426	-3,67
265,154669	264,14635	265,153626	-3,93
267,133934	266,12557	267,132846	-4,07
269,113199	268,10476	269,112036	-4,32
269,160817	268,15246	269,159736	-4,02
269,160817	268,1525	269,159776	-3,87
272,171716	271,16329	272,170566	-4,23
274,187366	273,17893	274,186206	-4,23
274,187366	273,17903	274,186306	-3,87
275,171382	274,16307	275,170346	-3,76
276,134268	275,12601	276,133286	-3,56
276,155398	275,14695	276,154226	-4,24
276,166631	275,15832	276,165596	-3,75
277,103029	276,09466	277,101936	-3,94
278,116905	277,1086	278,115876	-3,70
279,133934	278,12563	279,132906	-3,68
279,170319	278,16192	279,169196	-4,02
279,170319	278,16193	279,169206	-3,99
279,170319	278,16193	279,169206	-3,99

280,129183	279,12091	280,128186	-3,56
280,129183	279,12093	280,128206	-3,49
281,113199	280,10481	281,112086	-3,96
281,149584	280,1413	281,148576	-3,59
281,149584	280,14131	281,148586	-3,55
283,128849	282,12048	283,127756	-3,86
285,119347	284,11096	285,118236	-3,90
286,176133	285,1678	286,175076	-3,69
288,191783	287,1835	288,190776	-3,49
288,203016	287,19457	288,201846	-4,06
288,203016	287,19458	288,201856	-4,02
288,203016	287,19459	288,201866	-3,99
289,16188	288,15343	289,160706	-4,06
289,16188	288,15353	289,160806	-3,71
292,129183	291,1209	292,128176	-3,45
294,144833	293,13647	294,143746	-3,70
294,181218	293,17289	294,180166	-3,58
294,181218	293,17294	294,180216	-3,41
295,128849	294,12052	295,127796	-3,57
295,128849	294,1206	295,127876	-3,30
295,165234	294,15689	295,164166	-3,62
295,165234	294,15694	295,164216	-3,45
295,165234	294,15699	295,164266	-3,28
297,108114	296,09978	297,107056	-3,56
297,126741	296,1186	297,125876	-2,91
300,191783	299,18351	300,190786	-3,32
302,207433	301,19911	302,206386	-3,46
302,207433	301,19917	302,206446	-3,27
303,145167	302,13682	303,144096	-3,53
303,145167	302,13688	303,144156	-3,34
303,166297	302,15797	303,165246	-3,47
303,17753	302,16899	303,176266	-4,17
303,17753	302,16902	303,176296	-4,07
303,202682	302,19435	303,201626	-3,48
303,213915	302,20551	303,212786	-3,72
304,132555	303,12429	304,131566	-3,25
304,161546	303,15317	304,160446	-3,62
304,165568	303,15728	304,164556	-3,33
306,144833	305,1365	306,143776	-3,45
306,159438	305,15121	306,158486	-3,11
310,139748	309,13137	310,138646	-3,55
310,176133	309,16773	310,175006	-3,63
311,123764	310,11541	311,122686	-3,46
312,177864	311,16952	312,176796	-3,42
313,121656	312,11334	313,120616	-3,32
313,154669	312,14621	313,153486	-3,78
316,150313	315,14213	316,149406	-2,87
316,186698	315,17842	316,185696	-3,17
316,223083	315,21477	316,222046	-3,28
317,19318	316,18482	317,192096	-3,42

317,218332	316,21	317,217276	-3,33
318,165963	317,15751	318,164786	-3,70
318,181218	317,17289	318,180166	-3,31
318,181218	317,17291	318,180186	-3,24
318,181218	317,17292	318,180196	-3,21
318,202348	317,19397	318,201246	-3,46
318,202348	317,19402	318,201296	-3,31
319,140082	318,13176	319,139036	-3,28
320,145228	319,13687	320,144146	-3,38
320,181613	319,1732	320,180476	-3,55
320,181613	319,17325	320,180526	-3,39
322,187366	321,17896	322,186236	-3,51
322,187366	321,17904	322,186316	-3,26
329,149584	328,1411	329,148376	-3,67
329,149584	328,14111	329,148386	-3,64
330,202348	329,19392	330,201196	-3,49
330,238733	329,23029	330,237566	-3,53
331,197597	330,18918	331,196456	-3,45
331,197597	330,1892	331,196476	-3,38
331,197597	330,18922	331,196496	-3,32
331,233982	330,22554	331,232816	-3,52
332,181613	331,17316	332,180436	-3,54
332,217998	331,20956	332,216836	-3,50
332,217998	331,20957	332,216846	-3,47
332,217998	331,20958	332,216856	-3,44
332,217998	331,20961	332,216886	-3,35
333,155732	332,14728	333,154556	-3,53
333,192117	332,18356	333,190836	-3,84
333,213247	332,20475	333,212026	-3,66
334,139748	333,13128	334,138556	-3,57
334,176133	333,16782	334,175096	-3,10
336,140143	335,13167	336,138946	-3,56
336,191783	335,18343	336,190706	-3,20
336,191783	335,18343	336,190706	-3,20
338,182281	337,17371	338,180986	-3,83
338,182281	337,17393	338,181206	-3,18
340,197931	339,18954	340,196816	-3,28
342,156066	341,14772	342,154996	-3,13
343,197597	342,18924	343,196516	-3,15
344,254383	343,24602	344,253296	-3,16
344,254383	343,24604	344,253316	-3,10
345,144499	344,13608	345,143356	-3,31
345,213247	344,20481	345,212086	-3,36
345,213247	344,20492	345,212196	-3,04
345,22448	344,21599	345,223266	-3,52
345,22448	344,216	345,223276	-3,49
345,22448	344,21602	345,223296	-3,43
346,197263	345,18885	346,196126	-3,28
346,233648	345,22518	346,232456	-3,44
346,233648	345,22524	346,232516	-3,27

347,228897	346,22043	347,227706	-3,43
347,228897	346,22044	347,227716	-3,40
348,176528	347,16807	348,175346	-3,39
348,176528	347,16808	348,175356	-3,37
348,176528	347,16809	348,175366	-3,34
352,150313	351,14188	352,149156	-3,29
352,186698	351,17817	352,185446	-3,55
356,192846	355,1843	356,191576	-3,57
358,197263	357,18884	358,196116	-3,20
358,270033	357,26159	358,268866	-3,26
358,270033	357,2616	358,268876	-3,23
359,203745	358,19511	359,202386	-3,78
359,228897	358,2203	359,227576	-3,68
359,228897	358,2203	359,227576	-3,68
359,228897	358,22031	359,227586	-3,65
359,228897	358,22031	359,227586	-3,65
359,228897	358,22042	359,227696	-3,34
359,24013	358,23148	359,238756	-3,82
359,24013	358,23153	359,238806	-3,69
359,24013	358,23155	359,238826	-3,63
359,265282	358,25673	359,264006	-3,55
360,187761	359,1792	360,186476	-3,57
360,212913	359,20438	360,211656	-3,49
360,212913	359,2044	360,211676	-3,43
360,212913	359,2045	360,211776	-3,16
361,208162	360,19967	361,206946	-3,37
361,219395	360,21085	361,218126	-3,51
362,207433	361,19888	362,206156	-3,53
364,135058	363,12648	364,133756	-3,58
364,223083	363,21465	364,221926	-3,18
366,202348	365,19391	366,201186	-3,17
366,213581	365,20509	366,212366	-3,32
368,160483	367,15216	368,159436	-2,84
373,25578	372,24719	373,254466	-3,52
373,280932	372,27243	373,279706	-3,28
374,182281	373,17374	374,181016	-3,38
374,203411	373,19486	374,202136	-3,41
374,228563	373,22009	374,227366	-3,20
374,239796	373,23103	374,238306	-3,98
374,239796	373,23111	374,238386	-3,77
375,187427	374,17873	375,186006	-3,79
375,19866	374,19009	375,197366	-3,45
375,202682	374,19406	375,201336	-3,59
375,223812	374,21525	375,222526	-3,43
375,235045	374,22607	375,233346	-4,53
375,235045	374,22623	375,233506	-4,10
375,235045	374,22628	375,233556	-3,97
375,235045	374,22644	375,233716	-3,54
376,171443	375,16286	376,170136	-3,47
378,202348	377,19371	378,200986	-3,60

378,238733	377,23012	378,237396	-3,53
378,238733	377,23014	378,237416	-3,48
378,238733	377,23014	378,237416	-3,48
380,181613	379,17294	380,180216	-3,67
380,181613	379,17298	380,180256	-3,57
380,217998	379,20945	380,216726	-3,35
382,172111	381,16353	382,170806	-3,41
384,187761	383,17921	384,186486	-3,32
387,27143	386,26274	387,270016	-3,65
387,27143	386,26276	387,270036	-3,60
387,27143	386,26277	387,270046	-3,57
387,27143	386,26278	387,270056	-3,55
388,255446	387,24675	388,254026	-3,66
388,255446	387,24675	388,254026	-3,66
390,187093	389,17842	390,185696	-3,58
391,237354	390,22872	391,235996	-3,47
392,217998	391,20941	392,216686	-3,35
392,254383	391,24568	392,252956	-3,64
394,197263	393,18857	394,195846	-3,59
394,197263	393,18859	394,195866	-3,54
394,233648	393,22506	394,232336	-3,33
400,186698	399,17821	400,185486	-3,03
400,201303	399,19257	400,199846	-3,64
400,230294	399,22172	400,228996	-3,24
401,28708	400,27836	401,285636	-3,60
401,28708	400,27839	401,285666	-3,52
402,245944	401,2372	402,244476	-3,65
403,22996	402,22128	403,228556	-3,48
403,22996	402,22134	403,228616	-3,33
405,197992	404,18933	405,196606	-3,42
406,145623	405,13686	406,144136	-3,66
407,265282	406,25653	407,263806	-3,62
407,265282	406,25654	407,263816	-3,60
408,212913	407,20433	408,211606	-3,20
408,212913	407,20445	408,211726	-2,91
408,249298	407,24057	408,247846	-3,56
408,249298	407,24063	408,247906	-3,41
409,171777	408,16323	409,170506	-3,11
416,229231	415,22064	416,227916	-3,16
416,261594	415,25288	416,260156	-3,45
417,188095	416,17932	417,186596	-3,59
417,24561	416,23689	417,244166	-3,46
417,24561	416,23692	417,244196	-3,39
421,25578	420,24707	421,254346	-3,40
423,260197	422,25147	423,258746	-3,43
424,171443	423,1627	424,169976	-3,46
424,207828	423,19914	424,206416	-3,33
425,21431	424,20555	425,212826	-3,49
426,202348	425,19371	426,200986	-3,20
426,238733	425,23	426,237276	-3,42

427,197597	426,18906	427,196336	-2,95
431,265282	430,25661	431,263886	-3,24
431,265282	430,25662	431,263896	-3,21
433,20414	432,19545	433,202726	-3,26
438,234711	437,22604	438,233316	-3,18
441,249632	440,24102	441,248296	-3,03
442,197263	441,18857	442,195846	-3,20
444,304127	443,29529	444,302566	-3,51
444,304127	443,29537	444,302646	-3,33
450,245944	449,23683	450,244106	-4,08
450,245944	449,2372	450,244476	-3,26
451,22996	450,22123	451,228506	-3,22
451,266345	450,2577	451,264976	-3,03
453,209225	452,20046	453,207736	-3,29
462,198326	461,18954	462,196816	-3,27
466,277244	465,26855	466,275826	-3,04
467,224875	466,21612	467,223396	-3,17
469,208162	468,1994	469,206676	-3,17
485,250695	484,24179	485,249066	-3,36
499,200969	498,19213	499,199406	-3,13
